# Vessels encapsulating tumor clusters promote noninvasive metastasis of hepatocellular carcinoma by shaping an immunosuppressive microenvironment

**DOI:** 10.1172/JCI193758

**Published:** 2026-01-06

**Authors:** Bi-Yu Huang, Zheng-Qi Mi, Xiao-Yu Zhang, Yu-Chen Ji, Meng-Zhi Wu, Zi-Feng Cheng, Chen Xie, Shuai He, Jing Zhu, Jian-Hong Fang, Chong Wu, Bin-Kui Li, Yun-Fei Yuan, Limin Zheng, Shi-Mei Zhuang

**Affiliations:** 1 MOE Key Laboratory of Gene Function and Regulation, Guangdong Province Key Laboratory of Pharmaceutical Functional Genes, School of Life Sciences, State Key Laboratory of Oncology in South China, Sun Yat-sen University, Guangzhou, China.; 2 State Key Laboratory of Oncology in South China, Guangdong Provincial Clinical Research Center for Cancer and; 3 Department of Hepatobiliary Oncology, Sun Yat-sen University Cancer Center, Guangzhou, China.

**Keywords:** Hepatology, Immunology, Vascular biology, Liver cancer, Tregs

## Abstract

Vessels encapsulating tumor clusters (VETC), a distinct vascular pattern in hepatocellular carcinoma (HCC), facilitates noninvasive metastasis in whole clusters. The interaction between VETC and the tumor microenvironment requires exploration. Here, we found that, compared with human non-VETC-HCCs, VETC-tumors exhibited more PD1^+^CD8^+^ T cells and Tregs, especially TNFRSF4^+^ Tregs and Ki67^+^ Tregs, which showed increased immunosuppressive and proliferative activity. Such immunosuppressive status was also detected in tumor emboli of VETC-HCCs, and Treg density in emboli was positively associated with metastatic cell proliferation. VETC-HCCs revealed abundance correlation, closer spatial proximity, and stronger immunosuppressive ligand-receptor interactions between TNFRSF4^+^ Tregs/Ki67^+^ Tregs and PD1^+^CD8^+^ T cells. Depleting Tregs in mice reduced PD1^+^CD8^+^ T cells in primary lesions, tumor emboli, and metastatic foci of VETC-allografts, and attenuated allograft metastasis. TGF-β1 levels were upregulated in endothelial cells of VETC-HCCs and associated with TNFRSF4^+^ Tregs/Ki67^+^ Tregs enrichment. Disrupting VETC formation decreased endothelial TGF-β1 expression and reduced TNFRSF4^+^ Tregs, Ki67^+^ Tregs, PD1^+^CD8^+^ T cells, and Treg/CD8^+^ T cell ratios. Collectively, VETC may enhance Treg activity via TGF-β1, while Tregs promote and sustain CD8^+^ T cell exhaustion through immune inhibitory ligand-receptor interaction, thereby shaping the immunosuppressive microenvironment and enabling tumor clusters to retain such niche to disseminate. These findings disclose mechanisms of tumor immune microenvironment formation and provide rationales for precision medicine.

## Abstract 2

VETC HCCs exhibit stronger immunosuppressive microenvironment than Non-VETC HCCs. VETC promotes formation of immunosuppressive niche, enabling tumor emboli to carry such niche to facilitate metastasis.

## Graphical abstract

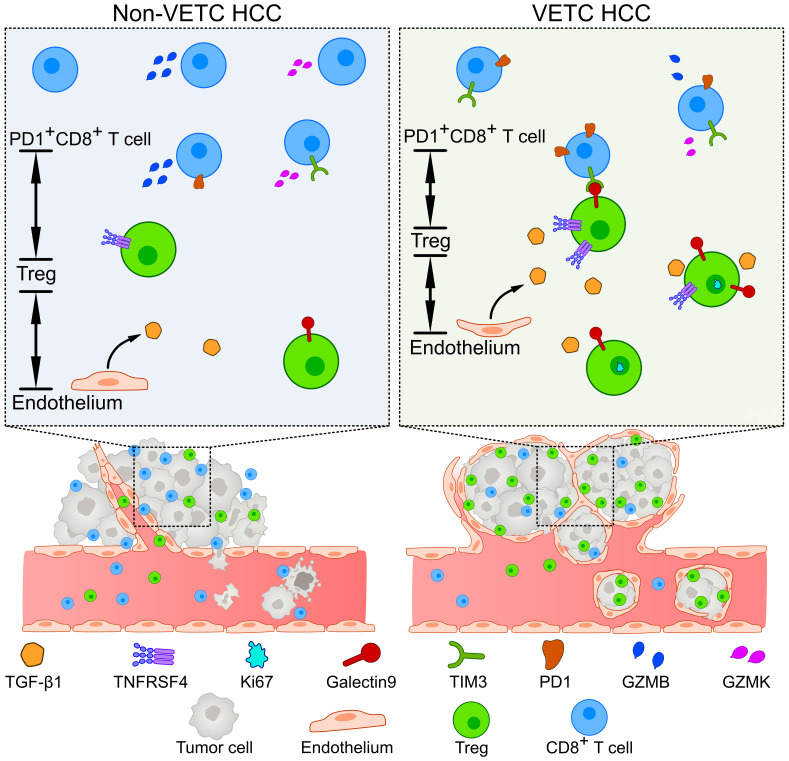

## Introduction

Hepatocellular carcinoma (HCC) is one of the most common malignancies worldwide, characterized by active angiogenesis, early metastasis, frequent recurrence, and high mortality (1). Recently, distinct vascular patterns have been identified in human HCC, including the classical capillary structures with discrete well-defined lumens and a novel pattern termed vessels encapsulating tumor clusters (VETC) (2–6). VETC consists of sinusoid-like vessels forming a cobweb-like network that isolates and surrounds individual tumor clusters (4–7). VETC exists in 35%–45 % of HCC cases and develops at an early stage of HCC (5–8). HCCs with discrete vessel lumens (designated as non-VETC HCCs) metastasize via an invasion-dependent route, whereas VETC enables the release of entire tumor clusters into the bloodstream in a migration/invasion–independent manner, resulting in higher metastasis/recurrence rate of VETC HCCs than non-VETC tumors (6–12). Furthermore, sorafenib therapy markedly prolongs the survival of patients with VETC HCC but provides little benefit for non-VETC HCC cases (13, 14). These findings underscore the distinct characteristics of VETC and non-VETC HCCs and highlight the importance of disclosing their difference for precision medicine.

Tumor immune microenvironment (TIME) plays important roles in tumor progression and treatment response via complex components and regulatory mechanisms (15–17). CD8^+^ T cells and Tregs play opposing roles in shaping the TIME. CD8^+^ T cells exert antitumor immunity by killing cancer cells (17–19), while immunosuppressive cells, in particular Tregs, empower tumors to evade immune surveillance by blocking CD8^+^ T cell activation and causing their exhaustion (20–22). A higher ratio of Treg/CD8^+^ T cells within the TIME is associated with worse prognosis in many cancer types (22–24). Tregs expressing tumor necrosis factor receptor superfamily (TNFRSF) members, such as TNFRSF4, TNFRSF9, and TNFRSF18, show high immune suppressive capacity and infiltrate in a range of malignancies (25–27). Recent studies suggest that endothelial cells may actively participate in immune modulation (28–31). Tumor endothelial cells (TECs) not only regulate immune cell infiltration through adhesion molecules but also express immunoregulatory mediators such as PD-L1 and TGF-β1, thereby shaping the immunosuppressive microenvironment (28, 32, 33). To date, the immune landscapes of VETC and non-VETC HCCs, the interaction between the vascular pattern and TIME, and the role of TIME in VETC-driven metastasis, remain to be explored.

In this study, we reveal that human VETC HCCs display a highly immunosuppressive TIME, characterized by enrichment of TNFRSF4^+^ Tregs, Ki67^+^ Tregs, and PD1^+^CD8^+^ T cells, compared with non-VETC tumors. VETC tumor emboli retain immunosuppressive niches resembling those of the primary tumor and exhibit increased proliferation compared to non-VETC HCC emboli. Mechanism investigations disclose that upregulation of TGF-β1 in the TECs of VETC HCCs fosters enrichment of Tregs with enhanced immunosuppressive and proliferative activity, then Tregs promote and sustain CD8^+^ T cell exhaustion in VETC tumors through immunosuppressive ligand-receptor interactions, like Galectin9-TIM3 axis. Disruption of VETC formation reduces endothelial TGF-β1 level and alleviates immunosuppression of the tumor microenvironment. These findings provide insights into the regulatory networks of the TIME and tumor metastasis from a perspective on the vascular pattern and may offer potential targets for the precision treatment of HCC.

## Results

### VETC HCC tissues exhibit a more immunosuppressive landscape than non-VETC HCCs.

**Figure 1:**
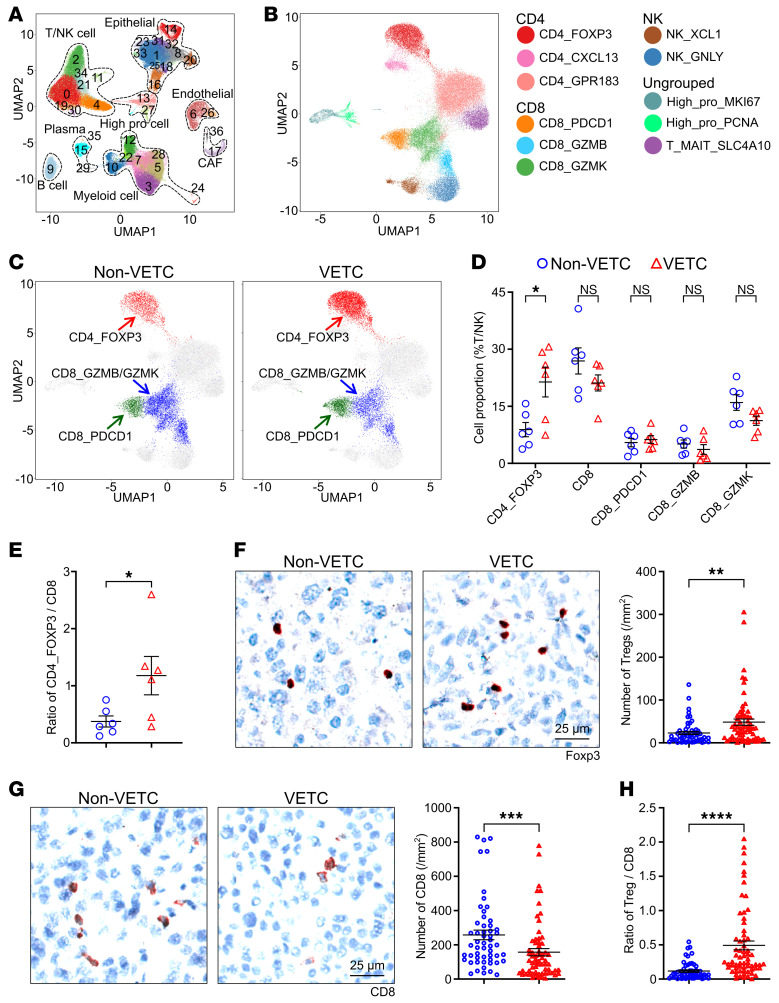
Single cell RNA-seq and IHC staining reveal an increase of Tregs and a decrease of CD8^+^ T cells in the tumor microenvironment of human VETC HCCs compared with non-VETC tumors. (**A**) Major cell types in HCC tumor microenvironment (TME). (**B**) The T/NK cell clusters. (**C**) The CD4_FOXP3 (red), CD8_GZMB/GZMK (blue) and CD8_PDCD1 (green) clusters. For **A**–**C**, cell clusters based on scRNA-seq data were visualized by using a UMAP plot. Each dot represents a single cell. (**D**) The proportions of CD4_FOXP3 and CD8 clusters in T/NK cells. (**E**) The ratio of CD4_FOXP3 cells to CD8^+^ T cells. For **A**–**E**, scRNA-seq data from 6 non-VETC and 6 VETC HCC tissues were analyzed. (**F** and **G**) The number of Tregs (**F**) and CD8^+^ T cells (**G**). (**H**) The ratio of Treg/CD8^+^ T cells. For **F**–**H**, 54 non-VETC tumors and 65 VETC tumors were analyzed by immunohistochemical staining. Scale bars: 25μm. *P < 0.05; **P < 0.01; ***P < 0.001; ****P < 0.0001, by 2-tailed Student’s *t* test (**D** and **E**) or Mann-Whitney *U* test (**F**–**H**). Data are shown as mean ± SEM.

To investigate whether the vascular pattern could affect the TIME in human HCCs, we first performed single-cell RNA-seq (scRNA-seq) analysis on non-VETC and VETC HCC tissues (Supplemental Figure 1, A–C, and Supplemental Tables 1 and 2; supplemental material available online with this article; https://doi.org/10.1172/JCI193758DS1). In total, 127,510 single cells were obtained from all examined tissues and categorized into 8 major clusters and 37 subsets based on canonical markers (Figure 1A, Supplemental Figure 2A, and Supplemental Table 3). All these cell types were presented in both non-VETC and VETC HCC tissues (Supplemental Figure 2, B and C), indicating a comprehensive and robust sampling of the tumor microenvironment. We then performed more detailed analysis on immune cells and endothelial cells, and showed that the proportions of CD4_FOXP3, B cells, and plasma cells differed between non-VETC and VETC tissues (Figure 1, B–D, and Supplemental Figure 3, A and B), whereas the proportion of other T/NK cell clusters, myeloid cell clusters, and endothelial cell clusters did not exhibit significant differences between these two HCC groups (Supplemental Figure 3, C–G).

Given the aim of this study and the dominance of T lymphocytes among immune cells and their primary antitumor function, we further analyzed 47,981 T/NK cells and identified clusters of CD4^+^ T cell, CD8^+^ T cell, NK cell, proliferating T cell, and mucosal-associated invariant T (MAIT) cell (Figure 1B, Supplemental Figure 3C, Supplemental Figure 4, and Supplemental Table 4). Among them, CD4_FOXP3 cells showed the most obvious difference between VETC and non-VETC HCCs (Figure 1, C and D, and Supplemental Figure 3C). The ratio of CD4_FOXP3 to CD8^+^ T cells was also much higher in VETC HCCs than in non-VETC tumors (Figure 1E). Subsequent immunohistochemical analysis on 119 clinical samples confirmed that VETC HCCs had more Tregs, fewer CD8^+^ T cells, and higher Treg/CD8^+^ T cell ratios, compared with non-VETC tumors (Figure 1, F–H). These distinct immune profiles between non-VETC and VETC HCCs were consistently observed in HCC subgroups stratified by tumor size, BCLC stage, and World Health Organization grading system for HCC (WHO grade) (Supplemental Figure 5, A–C), suggesting a stronger immunosuppression in the tumor microenvironment across VETC HCC subgroups.

### VETC tumors have more Tregs with enhanced proliferative and immunosuppressive activity and more CD8^+^ T cells with high exhaustion levels.

**Figure 2:**
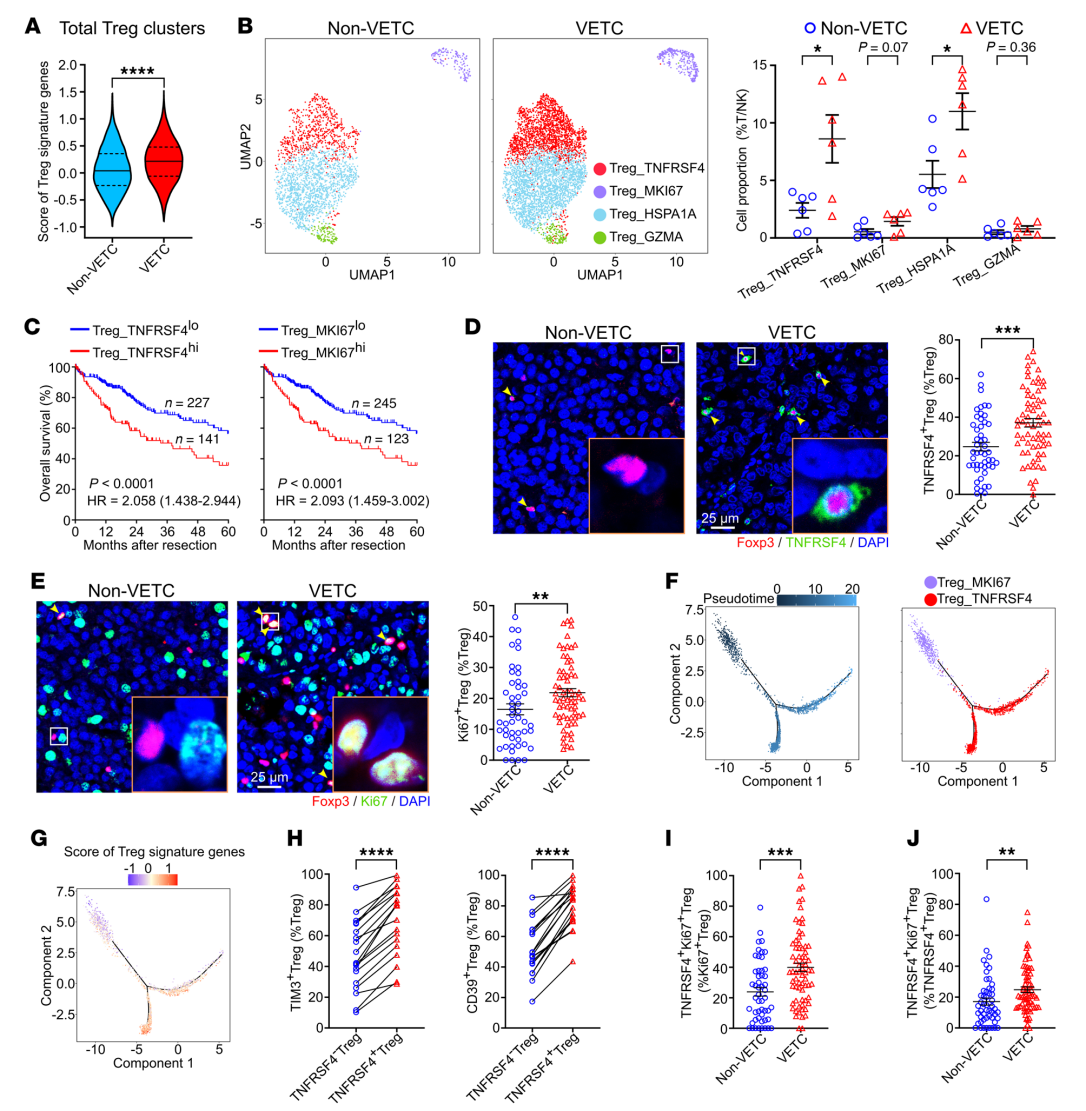
Tregs are more abundant and exhibit greater immunosuppressive and proliferative activity in human VETC HCCs compared with non-VETC tumors. (**A**) Score of tumor-specific Treg signature genes. The central line of violin plot denotes median value (50th percentile), the upper and lower lines denote the 75th and 25th percentiles, respectively. (**B**) UMAP plot visualization and quantification of four Treg clusters. non-VETC, VETC HCCs: *n* = 6, 6. (**C**) Kaplan-Meier overall survival analysis. TCGA HCC patients (*n* = 368) were stratified into high and low Treg_TNFRSF4 or Treg_MKI67 infiltration groups based on their signature genes in Supplemental Table 6. Infiltration levels were estimated by CIBERSORTx deconvolution on the RNA-seq data from TCGA. (**D** and **E**) Proportions of TNFRSF4^+^ Tregs and Ki67^+^ Tregs. Yellow arrows indicate TNFRSF4^+^ (**D**) or Ki67^+^ Tregs (**E**). (**F**) Pseudotime trajectory of Treg differentiation based on the signature genes of Treg_MKI67 and Treg_TNFRSF4. Genes are listed in Supplemental Table 6. Cells are assigned pseudotime scores, visualized as a gradient from dark to light blue, indicating progression from early to terminal states (left panel). (**G**) Score of tumor-specific Treg signature genes along the differentiation trajectory. For **A** and **G**, genes are listed in Supplemental Table 5. (**H**) The ratio of TNFRSF4^+^ Tregs and TNFRSF4^–^Tregs coexpressing TIM3 or CD39. *n* = 19. (**I** and **J**) The proportion of TNFRSF4^+^Ki67^+^ Tregs in Ki67^+^ Tregs (**I**) or TNFRSF4^+^ Tregs (**J**). non-VETC HCCs, *n* = 6 (**A** and **B**), 49 (**D** and **E**), 51 (**I** and **J**); VETC HCCs, *n* = 6 (**A** and **B**), 66 (**D**), 67 (**E**), 70 (**I** and **J**). scRNA-seq data were analyzed in **A**, **B**, **F**, and **G**, multiplex immunofluorescent staining in **D**, **E**, and **H**–**J**. Scale bars: 25μm. Data are shown as mean ± SEM.; *P < 0.05; **P < 0.01; ***P < 0.001; ****P < 0.0001, by Mann-Whitney *U* test (**A**, **E**, **I** and **J**), 2-tailed Student’s *t* test (**B**, **D**, and **H**), log-rank test (**C**).

We further examined Tregs in detail. Analysis on scRNA-seq data revealed higher expression of tumor-specific Treg signature genes in VETC tumor-derived Tregs (Figure 2A and Supplemental Table 5), compared with their non-VETC counterparts. Four Treg clusters, including Treg_TNFRSF4, Treg_MKI67, Treg_HSPA1A, and Treg_GZMA (Supplemental Figure 6A), were identified and the first 3 clusters were increased in VETC HCCs (Figure 2B). Among them, only high infiltration of Treg_TNFRSF4 or Treg_MKI67 cells, but not those of Treg_HSPA1A or Treg_GZMA cells, was associated with worse overall survival (Figure 2C, Supplemental Figure 6B, and Supplemental Table 6), thus, we focused subsequent investigations on Treg_TNFRSF4 and Treg_MKI67 clusters. The results showed that Treg_TNFRSF4 cells expressed the highest levels of immune inhibitory molecules among all clusters (Supplemental Figure 6C). And Treg_MKI67 cells from VETC HCCs revealed a markedly higher proliferative capacity (Supplemental Figure 6D), as indicated by increased expression of T-cell proliferation genes (34) (Supplemental Table 7). Multiplex immunofluorescent staining confirmed that the proportions of TNFRSF4^+^ Tregs and Ki67^+^ Tregs were substantially higher in VETC tumors, compared with non-VETC HCCs (Figure 2, D and E). To further delineate the relationship of Treg_TNFRSF4 and Treg_MKI67 cells, we performed pseudotime trajectory analysis and found that Treg_MKI67 cells progressively differentiated into Treg_TNFRSF4 cells, during which the expression of tumor-specific Treg signature genes gradually increased (Figure 2, F and G, and Supplemental Table 5). Furthermore, more TNFRSF4^+^ Tregs coexpressed immunosuppressive molecules, such as TIM3 and CD39, compared with TNFRSF4^–^ Tregs (Figure 2H), while no difference in the expression of these inhibitory molecules was observed between Ki67^–^ and Ki67^+^ Tregs (Supplemental Figure 6E). We further identified a transitional population coexpressing Ki67 and TNFRSF4 and showed that the proportion of Ki67^+^TNFRSF4^+^ Tregs was substantially higher in VETC tissues compared to non-VETC tissues (VETC versus non-VETC: 39.9% versus 23.9% of Ki67^+^ Tregs and 24.8% versus 17.1% of TNFRSF4^+^ Tregs) (Figure 2, I and J). These findings indicate that Tregs in VETC HCCs displayed enhanced proliferative and immunosuppressive activity.

**Figure 3:**
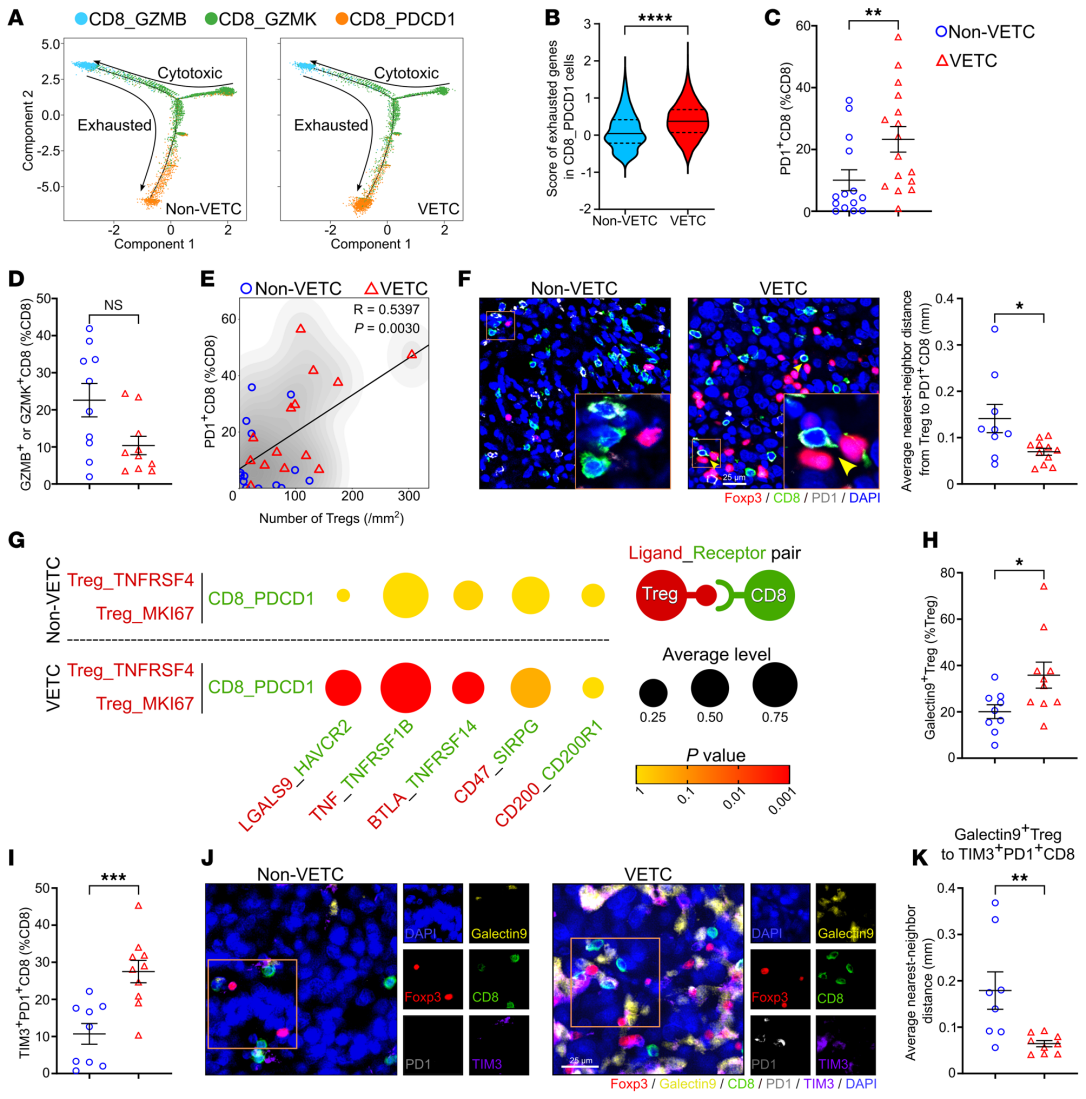
CD8^+^ T cells exhibit a more exhausted phenotype in human VETC tumors compared to non-VETC HCCs. (**A**) Pseudotime trajectory analysis on CD8^+^ T cell differentiation. (**B**) The score of exhausted genes in CD8_PDCD1 cells. Genes are listed in Supplemental Table 9. The central line of violin plot denotes median value (50th percentile), the upper and lower lines denote the 75th and 25th percentile, respectively. (**C**) The proportion of PD1^+^CD8^+^ T cells in CD8^+^T cells. (**D**) The proportion of GZMB^+^ or GZMK^+^CD8^+^ T cells in CD8^+^T cells. (**E**) Positive correlation between the number of Tregs and the proportion of PD1^+^CD8^+^ T cells in HCC tissues (*n* = 28). R represents the Pearson correlation coefficient. (**F**) Spatial proximity between Tregs and PD1^+^CD8^+^ T cells. Yellow arrows indicate close proximity of these cells. (**G**) CellPhoneDB analysis illustrated ligand-receptor interactions between Treg_TNFRSF4/Treg_MKI67 and CD8_PDCD1 cells. Dot size indicates average expression levels and color intensity denotes *P* value. (**H**) The proportion of Galectin9^+^ Tregs in Tregs. (**I**) The proportion of TIM3^+^PD1^+^CD8^+^ T cells in CD8^+^ T cells. (**J** and **K**) Spatial proximity between Galectin9^+^ Tregs and TIM3^+^PD1^+^CD8^+^ T cells. non-VETC HCCs, *n* = 6 (**A**, **B**, and **G**), 14 (**C** and **E**), 10 (**D**), 9 (**F**, **H**, and **I**), 8 (**K**); VETC HCCs, *n* = 6 (**A**, **B**, and **G**), 16 (**C**), 10 (**D**, **H**, and **I**), 14 (**E**),11 (**F**), 9 (**K**). scRNA-seq data were analyzed in **A**, **B**, and **G**, multiplex immunofluorescent staining in **C**–**F** and **H**–**K**. Scale bar: 25 μm. Data are shown as mean ± SEM. *P < 0.05; **P < 0.01; ***P < 0.001; ****P < 0.0001, by Mann-Whitney *U* test (**B**–**D**), 2-tailed Student’s *t* test (**F**, **H**, **I**, and **K**), Pearson’s correlation test (**E**).

We next performed a pseudotime trajectory analysis on scRNA-seq data to identify the developmental transitions of CD8^+^ T cells, and found a reduction of cytotoxic T cells (CD8_GZMB/GZMK) and an increase of exhausted T cells (CD8_PDCD1) in VETC tumors (Supplemental Figure 7, A and B, Figure 3A, and Supplemental Tables 8 and 9), indicating a pronounced shift toward an exhausted state. Compared with CD8_GZMB/GZMK cells, CD8_PDCD1 cells also revealed higher expression of other canonical exhaustion-associated molecules (like LAG3, HAVCR2, CTLA4, and TIGIT) and a higher exhaustion score (Supplemental Figure 7, C and D, and Supplemental Table 9). As shown, CD8_PDCD1 cells in VETC tissues exhibited a significantly higher exhaustion score than those from non-VETC samples (Figure 3B). These findings were corroborated by multiplex immunofluorescent staining, which confirmed an increased fraction of PD1^+^CD8^+^ T cells (Figure 3C and Supplemental Figure 7E) and a reduced proportion of GZMB^+^ or GZMK^+^CD8^+^ T cells (Figure 3D and Supplemental Figure 7F) in VETC tumors, compared with non-VETC HCCs. These data suggest that CD8^+^ T cells in VETC tumors may be more prone to immune dysfunction.

Considering that Tregs can promote the exhaustion and suppress the activation of CD8^+^ T cells (20–22), we evaluated the relationship between PD1^+^CD8^+^ T cells and Treg clusters in clinical samples. The proportion of PD1^+^CD8^+^ T cells was positively correlated with the number of total Tregs and with the fraction of either TNFRSF4^+^ Tregs or Ki67^+^ Tregs (Figure 3E and Supplemental Figure 7G). Examination of the spatial proximity revealed that the distance between Tregs and PD1^+^CD8^+^ T cells in VETC samples was much shorter than that in non-VETC samples (Figure 3F). Further cell-cell interaction analysis using CellPhoneDB software (35), focusing on immunosuppressive ligand-receptor pairs, revealed a significantly higher potential for interactions between Treg_TNFRSF4 or Treg_MKI67 and CD8_PDCD1 cells in VETC tumors, compared with non-VETC counterparts (Figure 3G). Among these ligand-receptor pairs, the LGALS9 (Galectin9)-HAVCR2 (TIM3) pair showed the most pronounced difference between non-VETC and VETC samples (Figure 3G). We therefore validated this interaction by multiplex immunofluorescent staining, and observed significantly higher proportions of Galectin9^+^Treg, Galectin9^+^TNFRSF4^+^ Treg, Galectin9^+^Ki67^+^Treg and TIM3^+^PD1^+^CD8^+^ T cells in VETC tissues than in non-VETC tissues (Figure 3, H–J, and Supplemental Figure 7, H and I). Moreover, the spatial distances from Galectin9^+^ Tregs, Galectin9^+^TNFRSF4^+^ Tregs, or Galectin9^+^Ki67^+^ Tregs to TIM3^+^PD1^+^CD8^+^ T cells were markedly shorter in VETC samples than in non-VETC samples (Figure 3, J and K, and Supplemental Figure 7, J and K), suggesting stronger immunosuppressive ligand-receptor interactions between Tregs and CD8^+^ T cells in VETC HCCs.

Taken together, in VETC tissues, enhanced enrichment and immunosuppressive activity of Tregs may promote and sustain CD8^+^ T cell exhaustion through ligand-receptor interactions, thereby shaping an immunosuppressive tumor microenvironment and dampening antitumor immunity.

### VETC HCC cells metastasize in clusters along with an immunosuppressive microenvironment to facilitate cell proliferation in tumor emboli.

**Figure 4:**
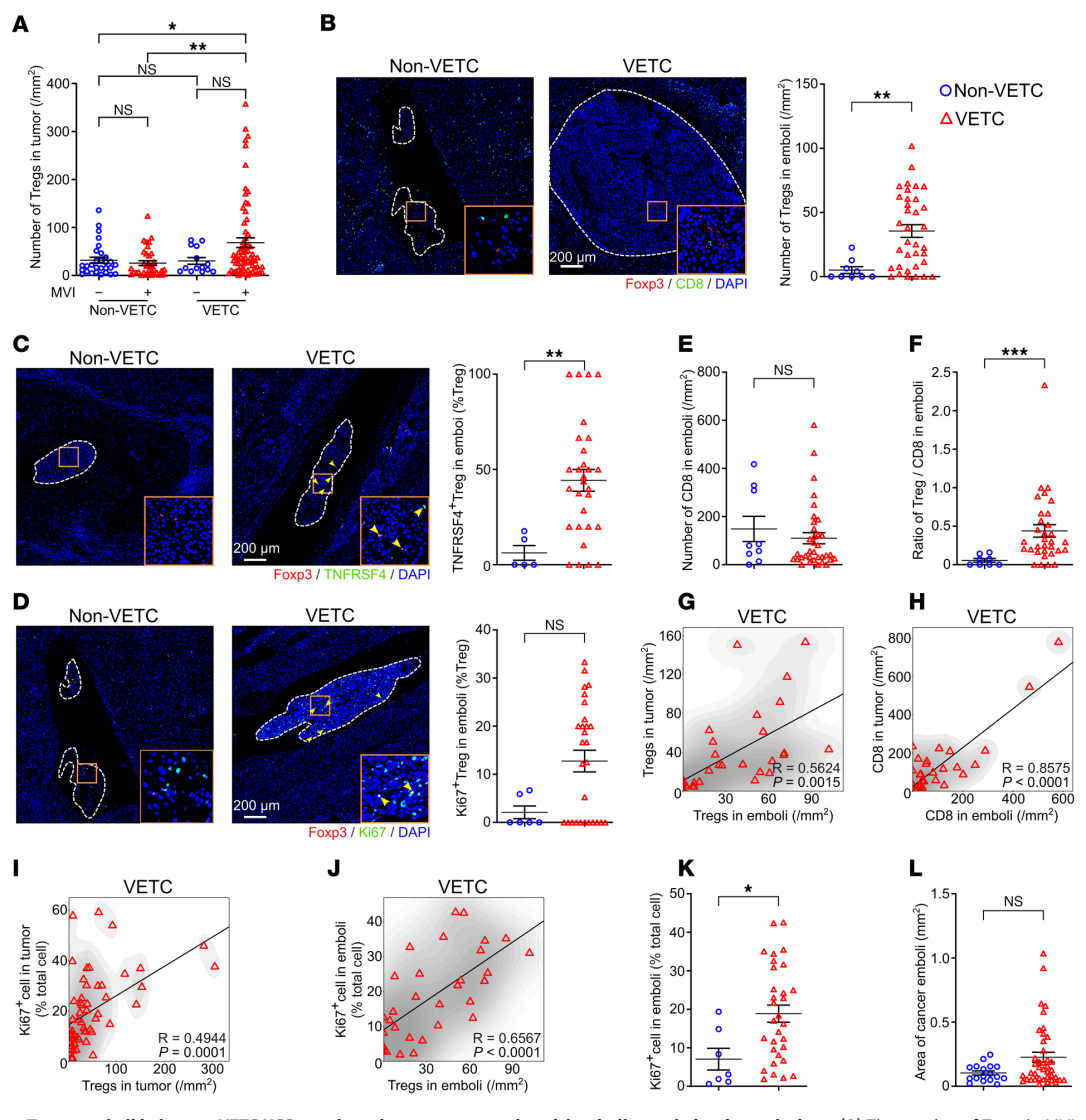
Tumor emboli in human VETC HCCs retain an immunosuppressive niche similar to their primary lesions. (**A**) The number of Tregs in MVI^+^ and MVI^–^ HCC tissues. MVI^+^ non-VETC, MVI^–^ non-VETC, MVI^+^ VETC, MVI^–^ VETC tumors: *n* = 35, 29, 69, 14. MVI^+^, presence of microvascular invasion. MVI^–^: absence of microvascular invasion. (**B**) The number of Tregs in tumor emboli. (**C** and **D**) The proportions of TNFRSF4^+^ Tregs (**C**) or Ki67^+^ Tregs (**D**) in tumor emboli. (**E**) The number of CD8^+^ T cells in tumor emboli. (**F**) The ratio of Treg/CD8^+^ T cells in tumor emboli. (**G** and **H**) Positive association between the number of Tregs (**G**) or CD8^+^ T cells (**H**) in the tumor emboli and in the primary tumor of VETC HCCs. (**I** and **J**) Positive association between the number of Tregs and the fraction of Ki67^+^ cells in both primary tumor (**I**) and tumor emboli (**J**) of VETC HCCs. For **G**–**J**, R represents the Pearson correlation coefficient. (**K**) The proportion of Ki67^+^ cells in tumor emboli. (**L**) The area of tumor emboli. For **B**–**H** and **J**–**L**, only the largest embolus was evaluated for those sections with more than one embolus. For **B**–**D**, white dashed lines outline tumor emboli. For **B** and **D**, representative images in non-VETC group, derived from adjacent sections of the same non-VETC HCC emboli with different stainings. non-VETC HCCs, *n* = 9 (**B** and **E**), 5 (**C**), 6 (**D**), 8 (**F**),7 (**K**), 17 (**L**); VETC HCCs: *n* = 35 (**B** and **E**), 29 (**C**, **G**, and **H**), 28 (**D**), 32 (**F**), 56 (**I**), 31 (**J** and **K**), 40 (**L**). Scale bars: 200 μm. Data are shown as mean ± SEM. *P < 0.05; **P < 0.01; ***P < 0.001, by 2-way ANOVA followed by Šidák’s post hoc test (**A**), Mann-Whitney *U* test (**B**–**F** and **L**), 2-tailed Student’s *t* test (**K**) and Pearson’s correlation test (**G**–**J**).

We have previously shown that VETC HCCs metastasize as a whole tumor cluster (6–8, 10). Notably, the increase of Tregs in the primary tumor was mainly detected in the VETC HCCs with microvascular invasion (MVI) (Figure 4A). We then explored whether the immunosuppressive microenvironment in primary tumors affected tumor emboli in adjacent nontumor liver tissues. Interestingly, abundant Tregs, TNFRSF4^+^ Tregs, and Ki67^+^ Tregs were detected within VETC tumor emboli, whereas these immune cells were rarely observed in non-VETC emboli (Figure 4, B–D). The number of CD8^+^ T cells was slightly lower (Figure 4E) and the ratio of Treg/CD8^+^ T cells was significantly higher within VETC tumor emboli (Figure 4F), suggesting enhanced immunosuppression.

Further investigations revealed that the numbers of both Tregs and CD8^+^ T cells in VETC tumor emboli were positively correlated with those in the primary tumors (Figure 4, G and H), while no such correlation was found in non-VETC HCCs (Supplemental Figure 8, A and B). Moreover, positive correlations between the density of Tregs and the proportion of proliferating cells (Ki67^+^) were observed in both primary sites and tumor emboli of VETC HCCs (Figure 4, I and J) but not in non-VETC HCCs (Supplemental Figure 8, C and D). As a result, the VETC tumor emboli had higher proliferation rates and larger sizes compared with non-VETC HCC emboli (Figure 4, K and L). These results indicate that the tumor emboli of VETC HCCs may carry an immunosuppressive niche from the primary site, thus possessing enhanced proliferation and survival capacity.

**Figure 5:**
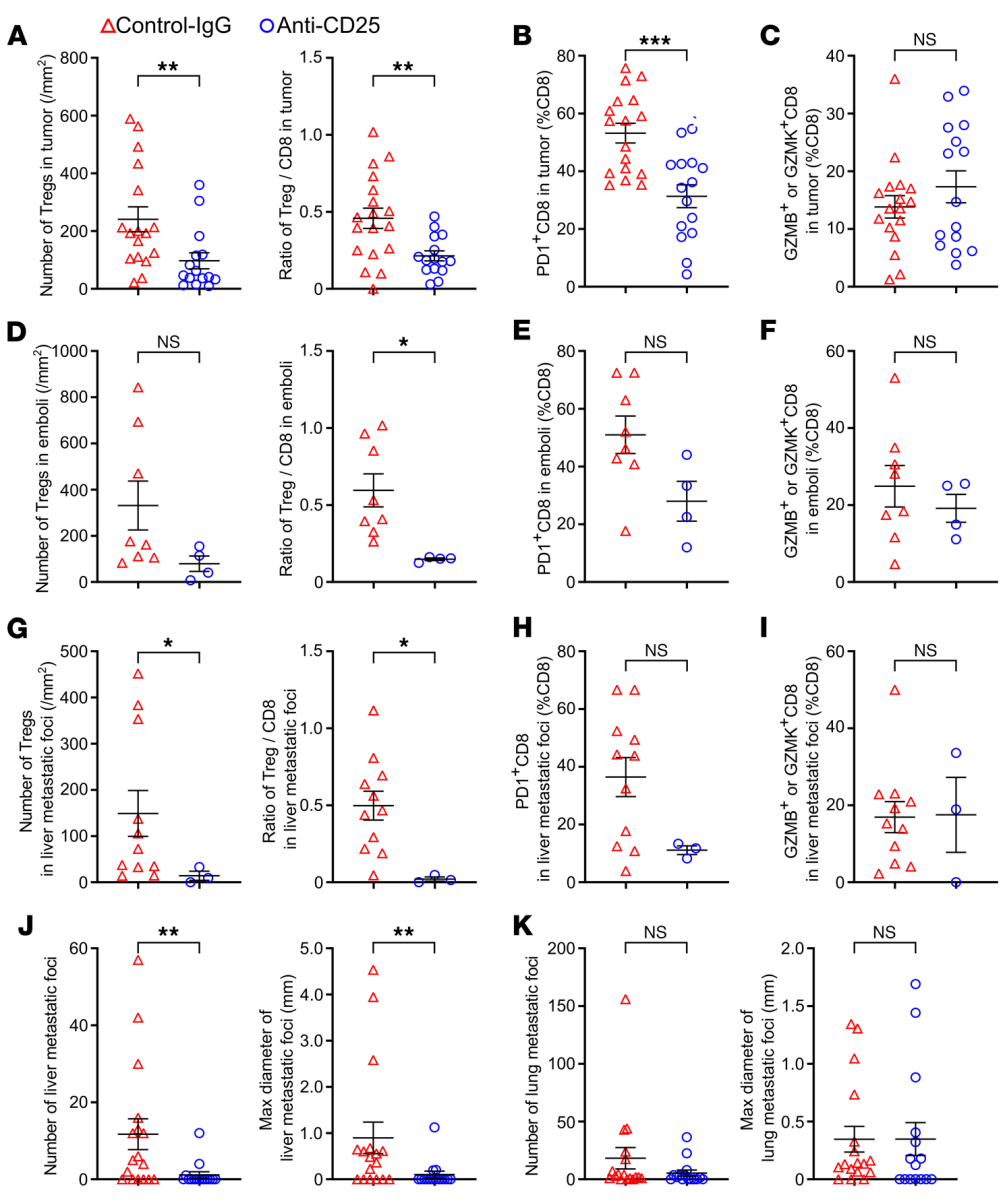
Depletion of Tregs by anti-CD25 antibody attenuates the immunosuppression and metastasis of mouse VETC hepatomas. (**A**–**C**) The effects of anti-CD25 antibody on the number of Tregs (**A**, left panel), the ratio of Treg/CD8^+^ T cells (**A**, right panel), the proportions of PD1^+^CD8^+^ T cells (**B**) and GZMB^+^/GZMK^+^CD8^+^ T cells (**C**) in the primary site of Hepa1-6 allografts. (**D**–**F**) The roles of anti-CD25 antibody on the number of Tregs (**D**, left panel), the ratio of Treg/CD8^+^ T cells (**D**, right panel), the proportions of PD1^+^CD8^+^ T cells (**E**) and GZMB^+^/GZMK^+^CD8^+^ T cells (**F**) in the tumor emboli of Hepa1-6 allografts. (**G**–**I**) The influences of anti-CD25 antibody on the number of Tregs (**G**, left panel), the ratio of Treg/CD8^+^ T cells (**G**, right panel), the proportions of PD1^+^CD8^+^ T cells (**H**) and GZMB^+^/GZMK^+^CD8^+^ T cells (**I**) in the liver metastatic foci of Hepa1-6 allografts. (**J**) The number (left panel) and maximal diameter of liver metastatic foci (right panel) of Hepa1-6 allografts. (**K**) The number (left panel) and maximal diameter of lung metastatic foci (right panel) of Hepa1-6 allografts. Hepa1-6 cells were implanted into murine liver. Control-IgG, isotype-matched control for anti-CD25 antibody. Control-IgG group, *n* = 17 (**A**–**C**, **J**, and **K**) or 8 (**D**–**F**) or 11 (**G**–**I**); Anti-CD25 group, *n* = 15 (**A**–**C**, **J**, and **K**) or 4 (**D**–**F**) or 3 (**G**–**I**). Data are shown as mean ± SEM. *P < 0.05; **P < 0.01; ***P < 0.001, by 2-tailed Student’s *t* test (**A**, right panel; **B**; **C**; **D**, right panel; **E**; **F**; **G**, right panel; **H**; **I**), Mann-Whitney *U* test (**A**, left panel; **D**, left panel; **G**, left panel; **J** and **K**).

We next investigated whether reversing the immunosuppression in the tumor could attenuate the metastasis of VETC HCCs. An anti-CD25 antibody was used to deplete Tregs in the mice implanted with Hepa1-6 hepatoma cells, which formed allografts with the VETC pattern (6, 8, 10). To avoid the influence of early Treg depletion on tumor growth (36), which may confound metastasis outcomes, anti-CD25 was administrated 10 days after tumor implantation, with a low dose and long interval (Supplemental Figure 9A). As shown, treatment with anti-CD25 effectively reduced the number of Tregs and the ratio of Treg/CD8^+^ T cells (Figure 5A and Supplemental Figure 9, B and C) and decreased the proportion of PD1^+^CD8^+^ T cells (Figure 5B), without significantly altering the number of total CD8^+^ T cells (Supplemental Figure 9, C and D) and the fractions of GZMB^+^ or GZMK^+^CD8^+^ T cells in primary tumors (Figure 5C). Consistent changes were observed in tumor emboli and liver metastatic foci (Figure 5, D–I, and Supplemental Figure 9, E–H). Lung metastatic foci were not analyzed for immune cells because they were too small for consecutive sections. While treatment with anti-CD25 did not affect primary tumor volume (Supplemental Figure 9I), it significantly reduced the incidence of tumor emboli (Control-IgG versus Anti-CD25: 8/17 versus 4/15, Table 1) and intrahepatic metastasis (Control-IgG versus Anti-CD25: 11/17 versus 3/15, Table 1), and reduced both the number and size of liver metastatic foci (Figure 5J and Supplemental Figure 9J). Additionally, anti-CD25 treatment also inhibited lung metastasis, although to a lesser extent (Figure 5K and Supplemental Figure 9K). These findings indicate that anti-CD25 may effectively deplete Tregs and thus relieve the immunosuppression in primary tumor, tumor emboli, and metastatic foci, leading to a systemic blocking in CD8^+^ T cell exhaustion and tumor metastasis.

Taken together, tumor emboli and metastatic foci of VETC HCCs harbor immunosuppressive niches, which may derive from the primary site and facilitate the survival and proliferation of metastatic tumor cells during circulation and colonization.

### VETC structure promotes the formation of an immunosuppressive tumor microenvironment.

**Figure 6:**
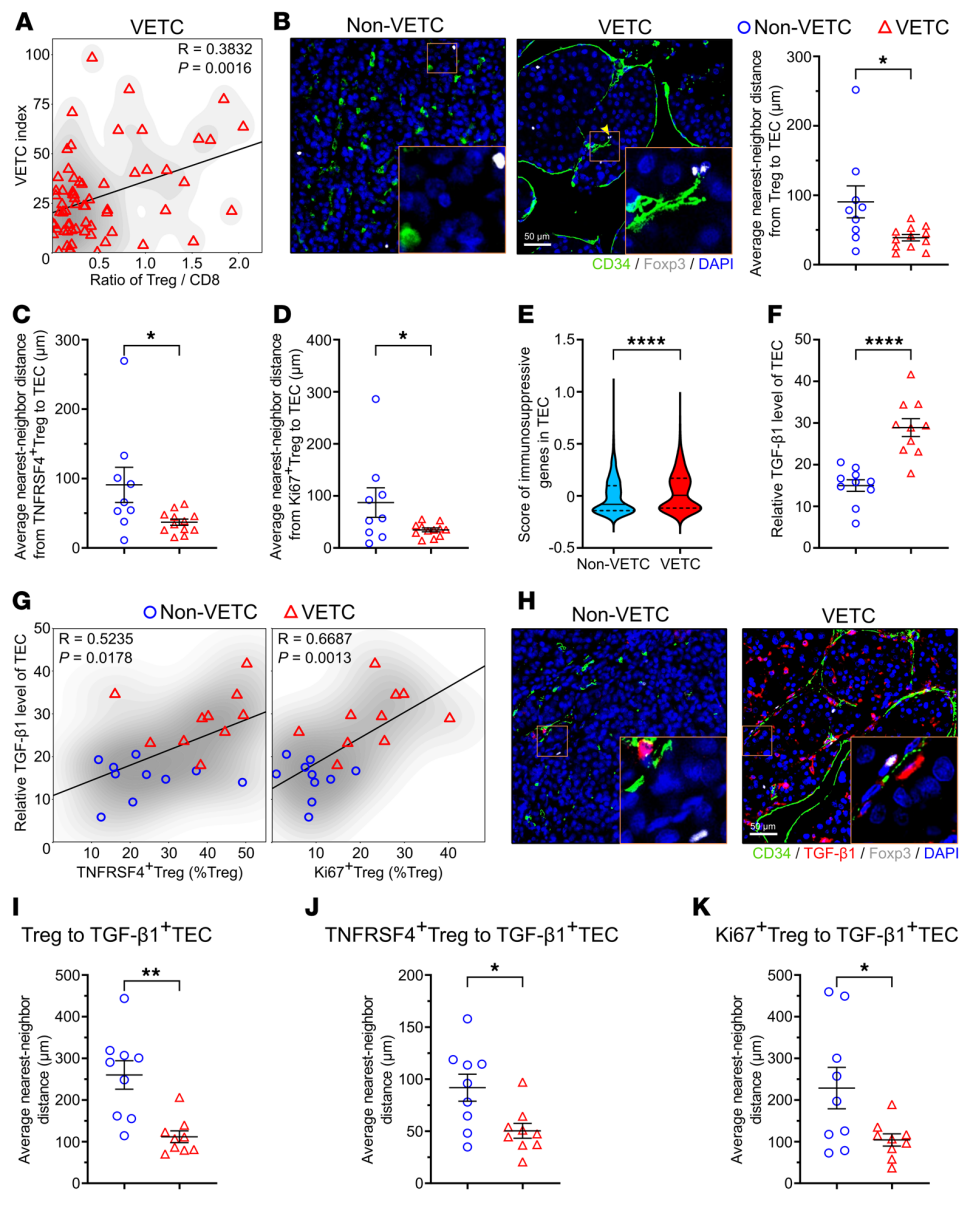
High TGF-β1 expression in TECs is associated with enhanced Treg enrichment and immunosuppressive/proliferative activities in human VETC HCCs. (**A**) Positive correlation between the VETC index and the ratio of Treg/CD8^+^ T cells in human VETC HCCs (*n* = 65). (**B**–**D**) Spatial proximity between Treg clusters and TECs. The distances from Tregs (**B**), TNFRSF4^+^ Tregs (**C**) or Ki67^+^ Tregs (**D**) to TECs in HCC samples are presented. Scale bars: 50 μm. (**E**) The score of immunosuppressive genes in TECs. The genes are listed in Supplemental Table 10. The central line of the violin plot denotes median value (50th percentile), the upper and lower lines denote the 75th and 25th percentile, respectively. (**F**) The relative levels of TGF-β1 in TECs. (**G**) Positive correlation between the proportion of TNFRSF4^+^ Tregs (left panel) or Ki67^+^ Tregs (right panel) and the TGF-β1 levels of TECs (*n* = 20). (**H**–**K**) The distances from Tregs (**H** and **I**), TNFRSF4^+^ Tregs (**J**), or Ki67^+^ Tregs (**K**) to TGF-β1^+^TEC. non-VETC HCCs, *n* = 9 (**B**–**D** and **I**–**K**), 6 (**E**), 10 (**F** and **G**); VETC tumors: *n* = 65 (**A**), 12 (**B**–**D**), 6 (**E**), 10 (**F** and **G**), 9 (**I**–**K**). Multiplex immunofluorescent stainings were performed for (**B**–**D**, and **F**–**K**), scRNA-seq data were analyzed in **E**, Scale bars: 50 μm. Data are shown as mean ± SEM. *P < 0.05; **P < 0.01; ****P < 0.0001, by Pearson’s correlation test (**A** and **G**), Mann-Whitney *U* test (**B**–**E**), 2-tailed Student’s *t* test (**F** and **I**–**K**).

We further explored the causal relationship between the VETC pattern and the immunosuppressive microenvironment. A strong positive correlation between the Treg/CD8^+^ T cell ratio and the VETC index was observed in VETC tumors (Figure 6A). We first examined whether the immune ecosystem influenced VETC formation. Depletion of Tregs with an anti-CD25 antibody in VETC tumor-bearing mice did not affect VETC formation and vessel area in tumors (Supplemental Figure 10A). Furthermore, both the VETC pattern and vessel area were similar in the Hepa1-6 allografts from C57BL/6J mice that are immunocompetent and from immunodeficient NSG mice lacking mature T, B, and NK cells (Supplemental Figure 10B), suggesting that lymphocytes within the tissue microenvironment do not affect VETC formation.

We next investigated whether and how VETC contributes to the formation of immunosuppressive microenvironment. Examination on the clinical samples revealed that Tregs were frequently localized near VETC (Figure 6B). Spatial proximity analysis further demonstrated that the distances of Tregs, TNFRSF4^+^ Tregs, or Ki67^+^ Tregs to tumor endothelial cells (TECs) were significantly shorter in VETC tissues than in non-VETC HCCs (Figure 6, B–D), indicating that VETC may have an influence on Tregs. Subsequent gene set scoring based on previously reported endothelial immunosuppressive signature genes (Supplemental Table 10) (28, 33) revealed that TECs in VETC tumors expressed higher levels of immunosuppressive genes than those in non-VETC samples (Figure 6E). Among these molecules, we focused on TGF-β1, due to its pivotal role in Treg proliferation, differentiation, and functional maintenance (37–39). As shown, the TECs of VETC tumors exhibited marked elevation of TGF-β1 expression compared with non-VETC TECs (Figure 6F). TGF-β1 levels in TECs were positively correlated with the proportions of TNFRSF4^+^ and Ki67^+^ Tregs (Figure 6G). Spatial proximity analysis also confirmed that the distance between these Treg subsets and TGF-β1^+^TECs was significantly shorter in VETC samples than in non-VETC samples (Figure 6, H–K). These data from human HCC samples indicate that high TGF-β1 expression in VETC TECs may cause enhanced enrichment and immunosuppressive activity of Tregs, thus highlighting the functional link between VETC and Tregs abundance/activity via TGF-β1.

**Figure 7:**
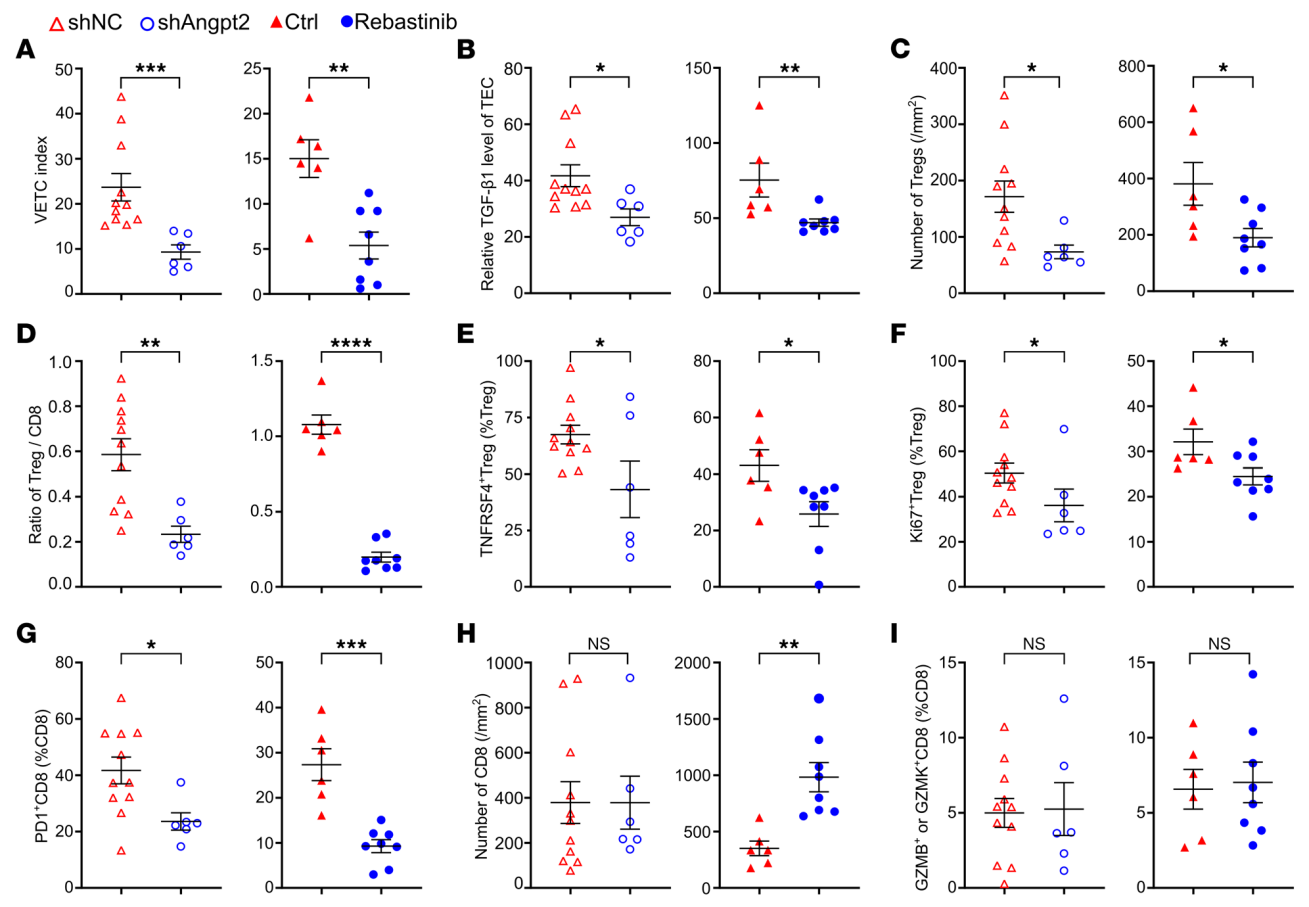
Disruption of VETC formation by inhibiting the Angpt2/Tie2 signaling relieves the immunosuppression in the tumor microenvironment. (**A**) Disruption of the Angpt2/Tie2 axis by shAngpt2 or Rebastinib disrupted VETC formation in Hepa1-6 allografts. Rebastinib, Tie2 inhibitor. (**B**) The levels of TGF-β1 in TECs. (**C**) The number of Tregs. (**D**) The ratio of Treg/CD8^+^ T cells. (**E** and **F**) The proportions of TNFRSF4^+^ Tregs (**E**) and Ki67^+^ Tregs (**F**). (**G**) The proportion of PD1^+^CD8^+^ T cells. (**H**) The number of CD8^+^ T cells. (**I**) The proportion of GZMB^+^ or GZMK^+^CD8^+^ T cells. For (**A**–**I**), left panels: shNC, Hepa-shNC allografts (negative control, *n* = 11); shAngpt2, Hepa-shAngpt2 allografts with stable silencing of Angpt2 (*n* = 6). Right panels: Ctrl, Hepa1-6 allografts in mice treated with control solution (*n* = 6); Rebastinib, Hepa1-6 allografts in mice treated with Rebastinib (*n* = 8). Data are shown as mean ± SEM. *P < 0.05; **P < 0.01; ***P < 0.001; ****P < 0.0001, by Mann-Whitney *U* test (**A**, left panel; **B**; **E** right panel; **F** left panel; **H** left panel), 2-tailed Student’s *t* test (**A**, right panel; **C**; **D**; **E**, left panel; **F**, right panel; **G**; **H**, right panel; **I**).

**Figure 8:**
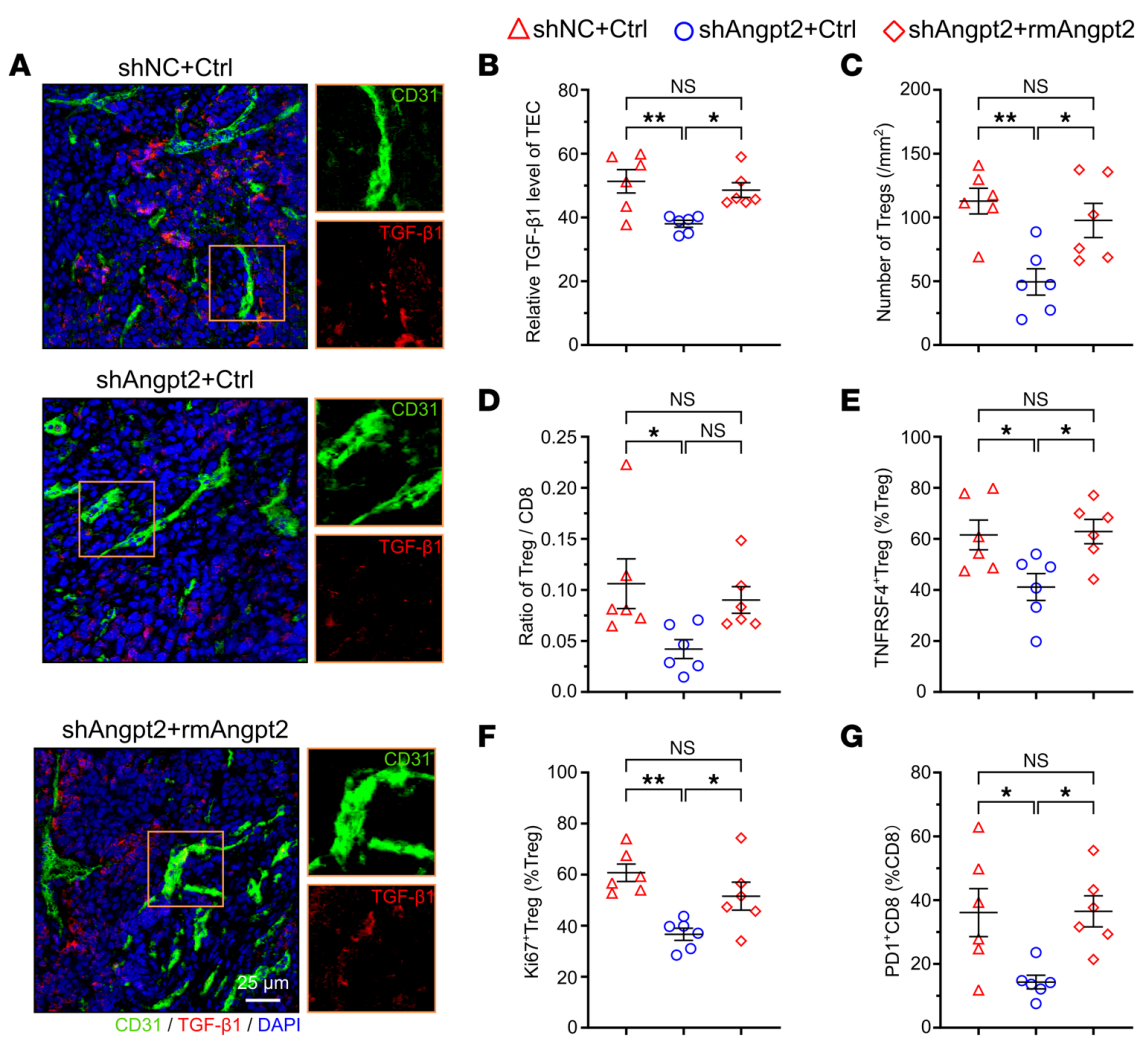
Restoration of VETC formation by recombinant Angpt2 supplementation reestablishes the immunosuppression in Hepa-shAngpt2 allografts. (**A** and **B**) Peritumoral injection with Angpt2 restored TGF-β1 expression in the TECs of Hepa-shAngpt2 allografts. (**C** and **D**) Angpt2 supplementation recovered the number of Tregs (**C**) and the ratio of Treg/CD8^+^ T cells (**D**) in Hepa-shAngpt2 allografts. (**E**–**G**) Angpt2 supplementation restored the proportion of TNFRSF4^+^ Tregs (**E**), KI67^+^ Tregs (**F**), and PD1^+^CD8^+^T cells (**G**) in Hepa-shAngpt2 allografts. For (**A**–**G**), shNC+Ctrl, Hepa-shNC allografts were peritumorally injected with 0.9% saline (negative control, *n* = 6); shAngpt2+Ctrl, Hepa-shAngpt2 allografts were peritumorally injected with 0.9% saline (*n* = 6); shAngpt2+rmAngpt2, Hepa-shAngpt2 allografts were peritumorally injected with recombinant mouse Angpt2 (*n* = 6). Scale bar: 25 μm. Data are shown as mean ± SEM. *P < 0.05; **P < 0.01, 1-way ANOVA followed by Tukey’s test (**B**–**G**).

It has been demonstrated that silencing Angpt2 in VETC HCC cells can inhibit VETC formation in mouse xenografts/allografts (6, 8, 10). We next explored the causative effect of VETC on shaping the immunosuppressive microenvironment by disrupting the VETC pattern via silencing Angpt2 (shAngpt2) in Hepa1-6 allografts or treating Hepa1-6 allografts with Tie2 inhibitor (Rebastinib) to block the Angpt2/Tie2 signaling (Supplemental Figure 11, A–C). Both interventions markedly inhibited VETC formation (Figure 7A and Supplemental Figure 11, D and E), downregulated endothelial TGF-β1 expression (Figure 7B and Supplemental Figure 11, F and G), and decreased the number of total Tregs (Figure 7C and Supplemental Figure 12, A and B) and the ratio of Treg/CD8^+^ T cells (Figure 7D), and reduced the proportions of TNFRSF4^+^ Tregs (Figure 7E and Supplemental Figure 12, A and B), Ki67^+^ Tregs (Figure 7F and Supplemental Figure 12, C and D), and PD1^+^CD8^+^T cells (Figure 7G and Supplemental Figure 12, E and F). Interestingly, the abundance of total CD8^+^ and cytotoxic CD8^+^ T cells was not affected by shAngpt2 (Figure 7, H and I, and Supplemental Figure 12G), but total CD8^+^ T cells was increased by Tie2 inhibitor (Figure 7H and Supplemental Figure 12H). Next, we performed a rescue experiment by peritumorally injecting recombinant Angpt2 into Hepa-shAngpt2 allografts (Supplemental Figure 13, A–C), and found that supplementation with Angpt2 restored VETC formation and endothelial TGF-β1 expression (Supplemental Figure 13D and Figure 8, A and B), raised the number of total Tregs and the ratio of Treg/CD8^+^ T cells (Figure 8, C and D), and recovered the proportions of TNFRSF4^+^ Tregs, Ki67^+^ Tregs, and PD1^+^CD8^+^T cells in shAngpt2-Hepa1-6 allografts (Figure 8, E–G, and Supplemental Figure 14, A–C). Total CD8^+^ and cytotoxic CD8^+^ T cells remained unaffected (Supplemental Figure 14, D and E). Collectively, these findings suggest that VETC structure may shape an immunosuppressive microenvironment by promoting the enrichment and immunosuppressive activity of Tregs and in turn CD8^+^ T cells exhaustion.

To rule out the possibility that shAngpt2 directly influenced the immunosuppressive microenvironment, Angpt2 was stably silenced in LC-Rd53 (Supplemental Figure 15A), a mouse hepatoma cell line that formed allografts with the non-VETC pattern (40). As shown, vessel area (Supplemental Figure 15B), the number of Tregs and CD8^+^ T cells, and the ratio of Treg/CD8^+^ T cells were not affected by silencing Angpt2 in LC-Rd53 allografts (Supplemental Figure 15, C–E). Consistently, the proportions of TNFRSF4^+^ Tregs, Ki67^+^ Tregs, PD1^+^CD8^+^, and GZMB^+^ or GZMK^+^CD8^+^ T cells were similar between LC-Rd53-shAngpt2 and LC-Rd53-shNC tumors (Supplemental Figure 15, F–I). These findings confirm that it is the disruption of VETC structure but not Angpt2 knockdown itself that alleviates the immunosuppression in the tumor microenvironment of VETC HCCs.

Collectively, these findings from both clinical samples and mouse allograft models highlight the functional link between VETC structure and the immunosuppressive microenvironment.

## Discussion

We and other research groups have demonstrated that VETC is a vascular pattern frequently observed in HCC tissues and a powerful predictor of HCC prognosis (2–7). We also revealed that non-VETC and VETC HCCs have marked differences in metastasis routes and in response to sorafenib and AR/RAC1 inhibitors (6, 8, 10, 13). VETC facilitates noninvasive metastasis of HCC cells in a cluster and thus increases metastatic efficiency (6–8, 10, 12). To date, it remains unexplored whether and how the TIME affects VETC-driven metastasis. Here, we showed that both primary sites and tumor emboli of human VETC HCCs displayed a highly immunosuppressive TIME, characterized by increased Tregs, TNFRSF4^+^ Tregs, Ki67^+^ Tregs,PD1^+^CD8^+^ T cells, and Treg/CD8^+^ T cell ratio. The abundance of Tregs was associated with the increase of cancer cell proliferation in the primary sites and tumor emboli of VETC HCCs. Notably, a positive correlation between the number of Tregs and CD8^+^ T cells was observed between primary sites and tumor emboli of VETC HCCs. Furthermore, depleting Tregs relieved immunosuppression in both primary lesions, tumor emboli, and metastatic foci and decreased metastasis of VETC allografts. These data indicate that the whole VETC tumor cluster carrying the immunosuppressive TIME from the primary site was released into the bloodstream, which confers metastatic cells with increased proliferation and survival potential during circulation and colonization in distant site. On the other hand, Tregs were rarely detected in the tumor emboli of non-VETC HCCs, and the area of tumor emboli in the non-VETC group was much smaller than that in VETC group, further supporting that non-VETC and VETC HCCs have markedly distinct characteristics that may influence tumor growth, metastasis, and therapy response. These findings suggest potential strategies for developing anti-HCC drugs and highlight that vascular pattern–based precision treatment should benefit patients with HCC.

Of note, the proportions of TNFRSF4^+^ Tregs and Ki67^+^ Tregs were positively correlated with PD1^+^CD8^+^ T cells. Spatial analysis showed that Tregs were located much closer to PD1^+^CD8^+^ T cells in VETC HCCs than in non-VETC tumors. Cell-cell interaction analysis further revealed stronger immunosuppressive ligand-receptor interactions between Tregs and PD1^+^CD8^+^ T cells in VETC HCCs, with the Galectin9-TIM3 pair showing the most prominent difference. An increase of Galectin9^+^ Tregs and TIM3^+^PD1^+^CD8^+^ T cells, and shorter spatial distances between them, were detected in VETC tissues compared with non-VETC HCCs. Therefore, enhanced enrichment and activity of Tregs in VETC HCCs may promote and sustain CD8^+^ T cell exhaustion through the Galectin9-TIM3 interaction.

Interestingly, although the number of CD8^+^ T cells is lower in human VETC HCCs compared with non-VETC tumors, disruption of VETC formation by silencing Angpt2 reduced Tregs and PD1^+^CD8^+^ T cells without increasing total CD8^+^ T cells in Hepa1-6 allografts, which may result from short experiment time, during which Treg-mediated exhaustion of CD8^+^ T cells precedes death of CD8^+^ T cells.

It is well known that cancer cells can affect the TIME. Emerging evidence indicates that TECs may also be involved in TIME remodeling (28–33). The adhesion of immune cells to TECs is a prerequisite for their migration across the vascular wall into tissues. It has been reported that the levels of cell adhesion molecules, such as E-selectin, ICAM1, and VCAM1 were downregulated in TECs, thus preventing T cell adhesion and infiltration into tumor tissues (28, 29, 41). TECs also exhibit high expression of immune checkpoint molecules, such as PD-L1, TIM3, and IDO, which suppress the function of CD8^+^ T cells (28, 32, 33). Vascular normalization has been shown to reverse immunosuppression in the tumor microenvironment, during which the vascular density either decreases or remains unchanged (42, 43). Therefore, the direct impact of vessel density on the TIME remains unclear. It is also unknown whether the vascular pattern, like VETC, affects the TIME. Herein, we observed a strong positive correlation between the VETC index and Treg/CD8^+^ T cell ratios in human VETC HCCs. Furthermore, Tregs were frequently localized around VETC TECs, and VETC TECs expressed higher levels of immunosuppressive genes, particularly TGF-β1, which was closely associated with Treg number and activity. Disruption of VETC formation reduced endothelial TGF-β1 and relieved immunosuppression in Hepa1-6 allografts, whereas Angpt2 replenishment restored VETC structure, TGF-β1 expression, and Tregs accumulation. These findings highlight the importance of the vascular pattern in shaping the immunosuppressive microenvironment in HCC.

It is worth noting that exhausted CD8^+^ T cells in our data simultaneously expressed cytotoxic molecules, a phenomenon corroborated by previous studies in HCC and across multiple cancer types (44, 45), suggesting that these cells exist in a “restrained activation” state that may retain cytotoxic transcriptional potential but exhibit limited killing capacity due to immunosuppressive signals and intrinsic exhaustion, which may be partially reversed by PD-1 blockade (45, 46). Based on these findings on the interplay between the vascular pattern and immune evasion, we propose that combining sorafenib or Angpt2 inhibitor with immune checkpoint regulators, like PD1/PD-L1 inhibitor or TNFRSF4 agonist, may be more beneficial for VETC HCCs than non-VETC cases. These schemes deserve further clinical study.

## Methods

### Sex as a biological variable.

Sex was not considered as a biological variable in this study. Human HCC samples analyzed in this study were obtained from both male and female patients, and no significant difference in sex distribution was observed between non-VETC and VETC HCC groups (Supplemental Table 11). Both male and female mice were used in mouse hepatoma allograft models, and similar findings were observed.

### Human tissue specimens.

Human HCC tissues and the matched adjacent nontumor liver tissues were collected from 152 patients who underwent HCC resection at Sun Yat-sen University Cancer Center between 2010 and 2022 and confirmed histologically. These patients did not receive any local or systemic anticancer treatments prior to surgery, and their clinical characteristics are summarized in Supplemental Tables 1, 2, and 11.

### Preparation of single-cell suspension for scRNA-seq.

Fresh tumor tissues from 6 non-VETC and 6 VETC HCC cases were immediately processed into single-cell suspensions using enzymatic digestion and mechanical dissociation. Briefly, tumor tissues were minced into approximately 1 mm^3^ pieces in DMEM (0-013-CVRC; Corning) supplemented with 10% FBS (10099141, Gibco), followed by digestion at 37°C for 50 min with constant shaking (80 rpm) in a mixture of DNase I (AMPD1-1KT, Sigma-Aldrich), dispase (354235, Corning), collagenase type II (17101015, Thermo Fisher) and collagenase type IV (17104019, Thermo Fisher), and then filtered through a 70 μm cell strainer to remove undigested tissues. The cell pellet was collected by centrifugation at 500*g* for 5 min, then resuspended in 1× Red Blood Cell (RBC) lysis buffer (C3702, Beyotine) and incubated on ice for 5 min to lyse red blood cells. After 2 washes with PBS, the lysed red cells were removed using a dead cell removal kit (130-090-101, Miltenyi Biotec) to enhance sample viability as follows: 100 μl of removal reagent was added per 10^7^ cells and incubated for 15 min at room temperature, followed by cell separation using a MACS buffer (PBS, 0.5% BSA, and 2 mM EDTA), a QuadroMACS separator (130-090-976, Miltenyi Biotec) and LS columns (130-042-401, Miltenyi Biotec). The column was pre-washed with 2 mL of MACS buffer prior to sample application. The cell suspension was then passed through the column and washed with 2 mL of MACS buffer. Viable cells were collected by centrifugation at 800*g* for 6 min at 4°C, yielding a minimum of 2 × 10^5^ viable cells per tissue sample.

### scRNA-seq and data processing.

scRNA-seq libraries were prepared using the Chromium platform (10x Genomics) following the manufacturer’s protocols. Briefly, single-cell suspensions were loaded into the Chromium Controller for droplet generation. After reverse transcription, emulsions were broken and barcoded cDNAs were purified using magnetic beads, followed by PCR amplification. Amplified cDNAs were used to construct single-cell 5′ gene expression libraries. For library construction, 50 ng of amplified cDNAs was fragmented, end repaired, and double-size selected using SPRIselect beads.

The prepared libraries were loaded onto individual lanes of the NovaSeq 6000 X system (Illumina) and sequenced with 150 bp paired-end reads. Raw sequencing data in Binary Base Call format were converted to FASTQ files using the bcl2fastq software (version 2.19.0.316, Illumina). Sequencing reads were aligned to reference genomes and feature-barcode matrices were generated using the Cell Ranger pipeline (version 6.0.1, 10X Genomics). Gene expression data was mapped to the human genome reference sequences (GRCh38) for further analysis.

### Quantification of single-cell gene expression levels and determination of cell types.

Gene expression matrices generated by Cell Ranger were imported into R workspace and converted into a Seurat object using the Seurat package (version 5.1.0). To ensure data reliability, we performed conventional quality control and also filtered out the potential impact of doublets and ambient RNA contamination. Only cells that had 100–800,000 UMIs (unique molecular identifiers) and expressed 500–25,000 genes with less than 25% mitochondrial gene content were selected for further analysis. Additionally, doublets were identified and removed using the DoubletFinder package, with an expected doublet rate of 0.07. Ambient RNA contamination was mitigated using the SoupX package. Cells passing these filters were classified as true single cells for further study.

Each of the 12 HCC single-cell sequencing datasets underwent an independent quality control and then merged for integrated analysis. We used NormalizeData and ScaleData functions in Seurat software to remove technical biases and ensure comparable gene expression levels across different cells. To correct for batch effects, the RunHarmony function was used to adjust for variations among samples. To visualize cellular composition and similarities in the data, dimensionality reduction and clustering were performed using the RunUMAP function.

Cell clusters were identified using the FindClusters function in Seurat software, based on the expression of known marker genes (Supplemental Table 3) for T/NK cells, B cells, plasma cells, myeloid cells, cancer-associated fibroblasts, endothelial cells, and malignant cells. The T/NK cell clusters and Treg clusters were further analyzed by specific marker genes (Supplemental Table 4 and Supplemental Table 6, respectively), using Seurat’s FindAllMarkers function to compare gene expression across clusters. Marker genes were identified with a Bonferroni-adjusted *P* value < 0.05 and a log_2_ fold change ≥ 0.6. Clusters with markers of multiple cell types were excluded. For each cluster, we assigned an identifier with a relevant marker gene (e.g., “CD4_FOXP3” for Tregs), ensuring strong specificity of gene expression.

### Calculation of functional module scores.

To assess functional differences within a specific cell cluster between VETC and non-VETC tissues, we calculated functional module scores for the target cell cluster using the AddModuleScore function in Seurat, which calculates a score for each cell based on the expression level of a specific gene set related to a biological process/function. The scores for tumor-specific Treg signature genes in Tregs (Supplemental Table 5) and T cell proliferation genes in Treg_MKI67 (Supplemental Table 7, respectively), and the scores for cytotoxic and exhausted CD8^+^ T cell genes (Supplemental Table 9) were calculated.

### Cell developmental trajectory.

The lineage trajectory of CD8^+^ T cells was constructed using Monocle2 (version 2.8.0). Cluster data from Seurat was directly input into Monocle2 for analysis. Next, we carried out density peak clustering (Monocle2 dpFeature procedure) to order cells based on the genes with differential expression between clusters (Supplemental Table 8). Significant genes (*P* < 0.01, |log_2_ foldchange| >1) ranked by log_2_ foldchange were used to order cells. Cell differentiation trajectory was then inferred through dimensionality reduction and cell ordering using Monocle2’s default parameters.

### Cellular ligand-receptor interaction analysis.

The ligand-receptor interactions between Treg clusters (Treg_TNFRSF4 and Treg_MKI67) and CD8_PDCD1 in HCC were analyzed using CellphoneDB software (version 5.0.1) to compare the expression of a receptor in 1 cell type and its corresponding ligand in another cell type. The average expression level was calculated by the average expression of each receptor-ligand pair across interacting cells. To assess significance, we permuted the cell type label 1,000 times and compared the observed mean to the randomly permuted mean. A significantly higher observed mean indicated a meaningful receptor-ligand interaction. Ligand-receptor pairs related to immune checkpoints were selected and their interaction potential between Treg clusters and CD8_PDCD1 in VETC and non-VETC tissues was visualized.

### Cell lines.

The mouse hepatoma cell line Hepa1-6 (ATCC CRL-1830) and human embryonic kidney (HEK) 293T cell line (ATCC CRL-3216) were purchased from Guangzhou Cellcook Biotech Co. Ltd. (Guangdong, China). Both cell lines were authenticated via short tandem repeat (STR) profiling. The primary mouse hepatoma cells (designated as LC-Rd53) were isolated from NrasG12V/sgp53-induced mouse liver tumors and established in our laboratory (40). Cells were cultured in DMEM supplemented with 10% FBS, 100 U/mL penicillin, and 100 μg/mL streptomycin, and maintained in a humidified atmosphere of 5% CO_2_ at 37°C.

### Establishment of cell lines with stable knockdown of Angpt2.

First, the shRNA expression plasmids (pCDH-shAngpt2, pCDH-shNC) were constructed. The fragments containing the shRNA sequences that targeted mouse Angpt2 (NM_007426.4) were generated by PCR and inserted between the *EcoRI* and *BamHI* sites in lentviral expression vector (pCDH-U6-MCS-EF1-copGFP-T2A-Puro), which was produced by replacing the CMV promoter of pCDH-CMV-MCS-EF1-copGFP-T2A-Puro (System Biosciences, Palo Alto, CA, USA) with U6 promoter (47). pCDH-shNC, which contained the shRNA sequence that was nonhomologous to any mouse genome sequence, was used as a negative control. All constructs were verified by direct sequencing. The sequences of shRNA targeting mouse Angpt2 or nonhomologous sequence are as follows: shAngpt2: 5′-GCT ATC CGT AAA GAA GAG C-3′; shNC: 5′-TGA ATT AGA TGG CGA TGT T-3′.

Next, lentiviral particles were prepared. HEK293T cells (1 × 10^6^) were seeded in a 100-mm plate for 24 h, then cotransfected with pCDH-shAngpt2 or pCDH-shNC and packaging plasmid (Lenti-X HTX Packaging Mix, Clontech, CA, USA) using Lipofectamine 3000 (L3000015, Thermo Fisher, CA, USA) and incubated at 37°C overnight, followed by replacing the medium with 10 mL DMEM containing 10% FBS and incubation at 37°C for an additional 48 h. The lentivirus supernatants were harvested by filtering through a 0.45 μm filter to remove cell debris.

Finally, Hepa1-6 and LC-Rd53 cells were infected with lentivirus supernatants diluted in DMEM with 10% FBS and 10 μg/mL polybrene for 12 h, then refreshed with 10% FBS-containing DMEM. The resulting stable lines, Hepa-shAngpt2 and LC-Rd53-shAngpt2, and their control lines Hepa-shNC and LC-Rd53-shNC were maintained in DMEM supplemented with 10% FBS and 0.25 μg/mL of puromycin.

The mRNA levels of Angpt2 were analyzed by real-time quantitative PCR. Total RNAs were extracted using TRIzol reagent (15596026, Thermo Fisher) and reverse transcribed using M-MLV reverse transcriptase (M1701, Promega). qPCR was performed on a LightCycler 480 (Roche Diagnostics, Germany) using 2× SYBR Green qPCR Master Mix (B21202, Bimake). β-Actin was used as a reference gene. All reactions were run in duplicate. The cycle threshold (Ct) values did not differ by more than 0.5 between the duplicates. The level of the target gene was normalized to that of β-actin, which yielded a value. The primer sequences for mouse Angpt2 amplification are as follows: sense strand: GAA CCA GAC AGC AGC ACA AA -3′; antisense strand: 5′- AGT TGG GGA AGG TCA GTG TG -3′. The primer sequences for mouse β-actin amplification: sense strand: CAT TGC TGA CAG GAT GCA GAA GG -3′; antisense strand: 5′- TGC TGG AAG GTG GAC AGT GAG G -3′.

### Mouse allograft models.

C57BL/6J mice (Strain NO.N000013) (3–4 weeks old) were purchased from GemPharmatech (Nanjing, China). NSG mice (Cat. NO. NM-NSG-001) (3–4 weeks old) were purchased from Shanghai Model Organisms Center, Inc (Shanghai, China). The mice were used for experiments at the age of 5 weeks.

For Treg depletion assay, Hepa1-6 cells (6 × 10^5^) were resuspended in 25 μL of Matrigel (3432-005-01, R&D Systems, MN, USA) /DMEM mixture (1:1 volume) and then inoculated into the liver of a C57BL/6J mouse. On days 10, 17, and 24 after implantations, mice were administrated with intraperitoneal injections (i.p.) of 200 μg anti-CD25 antibody in 100 μL 1 × PBS or an equivalent dose of anti-horseradish peroxidase IgG1 (isotype control) once a week 3 times. Tumors, livers, and lungs were collected 4 weeks after tumor cell inoculation. The tumor volume (V) was calculated using the formula V = (L × W^2^)/2, where L represents tumor length, and W stands for the tumor width. Antibodies used in this study are listed in Supplemental Table 12, along with their sources and clone numbers.

To compare the vascular pattern in C57BL/6J and NSG mice, Hepa1-6 cells (6 × 10^5^) were resuspended in 25 μL of Matrigel/DMEM (1:1 volume) and then inoculated into mouse livers. Tumors were dissected 4 weeks after inoculation.

For the liver orthotopic allograft model with stable silencing of Angpt2, Hepa-shAngpt2, LC-Rd53-shAngpt2, and their control lines (1 × 10^6^) were resuspended in 25 μL of Matrigel/DMEM (mixed at 1:1 volume ratio) and then inoculated into the liver of C57BL/6J mice. Tumors were dissected 4 weeks after inoculation.

For the liver orthotopic allograft model with treatment of Tie2 inhibitor, Hepa1-6 cells (3 × 10^5^) were resuspended in 25 μL of Matrigel/DMEM mixture (1:1 volume) and inoculated into the liver of C57BL/6J mice. Starting on day 10 after inoculation, mice received daily i.p. injections of Rebastinib (20 mg/kg, HY-13024, MedChemExpress) or control solution (40% PEG300, 53% saline, 5% Tween-80, and 2% DMSO) until day 21.

For subcutaneous allograft model with Angpt2 supplementation, Hepa-shNC or Hepa-shAngpt2 cells (5 × 10^5^) were resuspended in 100 μL of Matrigel /DMEM mixture (1:3 volume) and inoculated s.c. into both hind limbs of C57BL/6J mice. Starting on day 7 after inoculation, recombinant mouse Angpt2 protein (0.2 μg per 100 mm^3^ of tumor volume, 7186-AN-025, R&D Systems) or control solution (0.9% saline) were peritumorally injected into Hepa-shAngpt2 allografts every other day until day 15.

### IHC and multiplex IF staining.

Frozen HCC tissues embedded in optimal cutting temperature (OCT, 4583, SAKURA Finetekn) compound were sectioned at 8 μm, fixed in 4% paraformaldehyde (PFA) for 10 min, and incubated with primary antibodies at 37°C for 30 min. Formalin-fixed, paraffin-embedded tissues were sectioned at 4 μm, deparaffinized in xylene, rehydrated through a graded ethanol series, and then treated with 0.3% hydrogen peroxide to quench endogenous peroxidase activity. Antigen retrieval was performed by pressure cooking in 10 mM citrate buffer (pH 6.0), followed by overnight incubation with primary antibodies at 4°C.

For IHC staining, a 2-step Dako EnVision System (K5007, Dako Denmark A/S) was used for both human and murine antigens to detect protein expression, followed by hematoxylin (DH0005, Leagene) staining. Images were scanned by Leica Aperio VERSA 200 (Leica Biosystems) at 20 × magnification.

For multiplex IF staining, different primary antibodies were sequentially applied, followed by incubation with horseradish peroxidase-conjugated secondary antibody and signal amplify by Vari Fluor 488 TSA (Tyramide Signal Amplification) or Vari Fluor 555 TSA or Vari Fluor 640 TSA (HY-D1837, HY-D1836 and HY-D18364, MedChemExpress). Nuclei were counterstained with 4’,6-diamidino-2phenylindole (DAPI, D9542, Sigma-Aldrich).

For IHC and multiplex IF staining, detailed information regarding antibody sources and catalog numbers is provided in Supplemental Table 12.

### Evaluation of staining signals and cell-cell distance.

The stained sections were scanned using Aperio VERSA Digital Pathology Scanner (Leica Biosystems) or high-resolution confocal microscopy (Leica TCS SP8X, Leica Biosystems), and analyzed with QuPath (48), where tumor regions and staining thresholds were defined by 2 researchers, who were blinded to sample’s characteristics. Cell detection and classification functions in QuPath were applied to segment the image and quantify signal-positive or negative cells.

For multiplex IF staining, the image was first segmented into individual cell regions and split into layers corresponding to different antibody stains, based on fluorescence wavelength. A positive signal threshold was set for each antibody layer, based on signal location and intensity. A cell was considered double positive if it showed 2 signals exceeding the positive threshold at the same location.

The distance between nearest neighbor cells was calculated using ImageJ (49). The cell centroid coordinate of each Foxp3^+^Treg cell and PD1^+^CD8^+^ T cell was recorded. A distance matrix was then generated for each Treg cell, and the nearest PD1^+^CD8^+^ T cell was identified. The shortest distance for each cell was used for subsequent spatial proximity analysis. Similar procedures were applied for all nearest-neighbor cell-cell distance evaluation in this study.

To evaluate metastasis, 30 serial sections from the livers and lungs of mouse allograft model were stained with H&E, then scanned by Leica Aperio VERSA 200 (Leica Biosystems) at 20 × magnification and independently reviewed under a microscope by 2 researchers who were blinded to the treatment. The metastasis rate was defined as the proportion of tumor-bearing mice with detectable metastatic foci. For each mouse, the total number of metastatic foci across the 30 serial sections and the average diameter of the largest foci in each section were calculated.

To evaluate the VETC index and vessel area in mouse orthotopic liver allografts and human HCC tissues, the sections were subjected to IHC staining for mouse CD31 and human CD34, then scanned by Leica Aperio VERSA 200 (Leica Biosystems) at 20× magnification. The vascular pattern of each HCC tissue was determined as described (6), independently by 2 researchers who were blinded to sample’s information. The VETC pattern was defined as vessels forming cobweb-like networks that encapsulated individual tumor clusters. The cases with a visible VETC pattern in the whole or part of tumor section were identified as VETC HCC/tumor/emboli, and those without any VETC pattern in the whole tumor section were identified as non-VETC HCC/tumor/emboli. The VETC index and vessel area were assessed as reported (6). In brief, a VETC index was used to quantify the extent of VETC formation. The 5 most intensely vascularized fields were identified, and the number of individual tumor clusters that were surrounded by endothelium for at least 75% (human samples) or 50% (mouse allografts) of the tumor surface was recorded. The average number of endothelium-coated tumor clusters per field was presented as VETC index. To determine the vessel area in each allograft and human HCC tissues, mouse CD31 or human CD34 staining area in the 5 most vascularized fields were analyzed using ImageJ. The CD31- or CD34-staining area relative to the total tumor area indicated relative vessel area.

The expression levels of Angpt2 in mouse allografts, indicated by the histological score (H-score), was evaluated using Qupath software, according to the following formula: *H_score_* = ∑*P_i_*(*i* +1), where *i* represents the intensity score, categorized as no staining, scored as 0; weak staining, scored as 1; moderate staining, scored as 2; or strong staining, scored as 3 staining, and *P_i_* stands for the percentage of positively stained cells, ranging from 0–100. The expression of TGFβ1 in TECs was assessed by quantifying immunofluorescence signals with ImageJ. The integrated density of TGFβ1 immunofluorescence within CD31^+^ (mouse) or CD34^+^ (human) areas was measured. This value was then divided by the total pixel area of the corresponding CD31/CD34-staining region to obtain a relative expression level of TGFβ1.

### Statistics.

Survival of patients was analyzed using Kaplan-Meier Plotter based on the transcriptome profiling data from The Cancer Genome Atlas (TCGA). Cell cluster infiltration levels were estimated by CIBERSORTx (50) deconvolution of RNA-seq data from TCGA. Hazard ratios (HRs) and 95% confidence intervals (CIs) were calculated using Cox proportional hazards regression models.

Data were expressed as the mean ± SEM. The Shapiro-Wilk method was used to test normal distributions. If data met normality, 2-tailed Student’s *t* test was applied; otherwise, Mann-Whitney *U* test was used to compare the differences between 2 groups. One-way ANOVA was applied to compare the means of a dependent variable across 3 or more groups and evaluate the impact of 1 independent variable, while 2-way ANOVA was used when 2 independent variables were involved. All statistical tests were 2-sided and *P* < 0.05 was considered statistically significant. Analyses were performed with GraphPad Prism (version 8.0, GraphPad Software).

### Study approval.

For experiments using human samples, written informed consent was obtained from each patient, and the protocol was approved by the Institutional Research Ethics Committee of the Sun Yat-sen University Cancer Center (Guangzhou, China) (HCSW-2019-001). The patients were anonymously coded in accordance with ethical guidelines, as instructed by the Declaration of Helsinki.

All animal experiments were performed following the Guide for the Care and Use of Laboratory Animals (National Institutes of Health publication nos. 80-23, revised 1996) and the institutional ethical guidelines. The study protocol was approved by the Institutional Animal Care and Use Committee at Sun Yat-sen University (SYSU-IACUC-2022-B00146).

### Data availability.

The raw scRNA-seq data were deposited in the Genome Sequence Archive (GSA-Human) (51) in National Genomics Data Center (52). The scRNA-seq data accession number is HRA015108. The data points in graphs are provided in the file of Supporting Data Values. Requests for any other data should be directed to and will be fulfilled by the corresponding author.

## Author contributions

BYH, ZQM, and XYZ: Conceptualization, study design, experiment operation, data analysis and manuscript writing. The order of co-first authors was determined by their relative contributions. YCJ, MZW, ZFC, and CX: study design, experiment operation, data analysis. SH and JZ: Experiment operation, data analysis. JHF and CW: study design, data interpretation. BKL and YFY: collection of human tissues and clinical information, data interpretation. LZ and SMZ: Conceptualization, supervision, study design, data interpretation, funding acquisition, project administration, manuscript writing.

## Funding support

- National Key R&D Program of China (2022YFA1303302).
- Noncommunicable Chronic Diseases-National Science and Technology Major Project (2025ZD0544400).
- National Natural Science Foundation of China (82230093, 82488101, 32230034, 32100573).
- Guangdong Basic and Applied Basic Research Foundation (2023A1515012478).
- Hunan Provincial Natural Science Foundation of China (2023JJ40920).

## Supplementary Material

Supplemental data

Supplemental table 1

Supplemental table 2

Supplemental table 3

Supplemental table 4

Supplemental table 5

Supplemental table 6

Supplemental table 7

Supplemental table 8

Supplemental table 9

Supplemental table 10

Supplemental table 11

Supplemental table 12

Supporting data values

## Figures and Tables

**Table 1:**
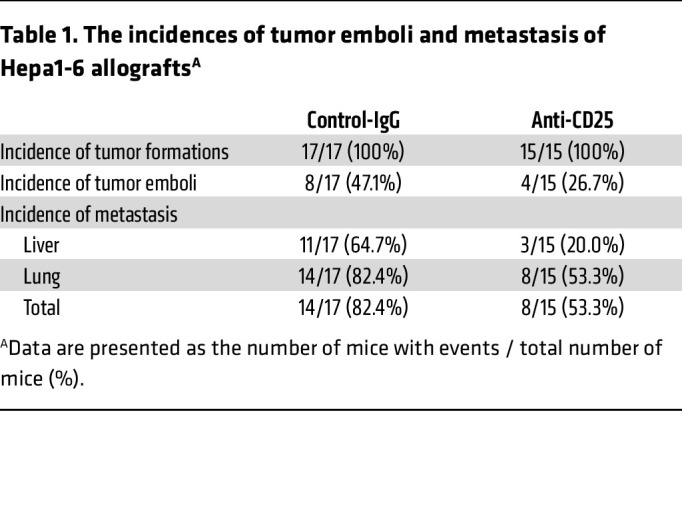
The incidences of tumor emboli and metastasis of Hepa1-6 allografts^A^

## Version 1 01/06/2026

In-Press Preview

## Version 2 02/16/2026

Electronic publication
