# Supplementary material for: Vessels encapsulating tumor clusters promote noninvasive metastasis of hepatocellular carcinoma by shaping an immunosuppressive microenvironment
Source: J Clin Invest. 2026 Jan 6;136(4):e193758. doi: 10.1172/JCI193758 (PMC12904702; doi:10.1172/JCI193758)
Supplement: Supplemental data [file jci-136-193758-s332.pdf]

Supplementary Materials for

Vessels encapsulating tumor clusters promote non-invasive metastasis of hepatocellular carcinoma by shaping an immunosuppressive microenvironment

Bi-Yu Huang[1#], Zheng-Qi Mi[1#], Xiao-Yu Zhang[1#], Yu-Chen Ji[1], Meng-Zhi Wu[1], Zi-Feng Cheng[1], Chen Xie[1], Shuai He[2], Jing Zhu[1], Jian-Hong Fang[1], Chong Wu[1], Bin-Kui Li[3], Yun-Fei Yuan[3], Limin Zheng[1*], Shi-Mei Zhuang[1*]

[1]MOE Key Laboratory of Gene Function and Regulation, Guangdong Province Key Laboratory of Pharmaceutical Functional Genes, School of Life Sciences, State Key Laboratory of Oncology in South China, Sun Yat-sen University, Guangzhou, P. R. China.

[2]State Key Laboratory of Oncology in South China, Guangdong Provincial Clinical Research Center for Cancer, Sun Yat-sen University Cancer Center, Guangzhou, P. R. China.

[3]Department of Hepatobiliary Oncology, Sun Yat-sen University Cancer Center, Guangzhou, P. R. China

This PDF file includes:

Supplemental Figures 1 to 15

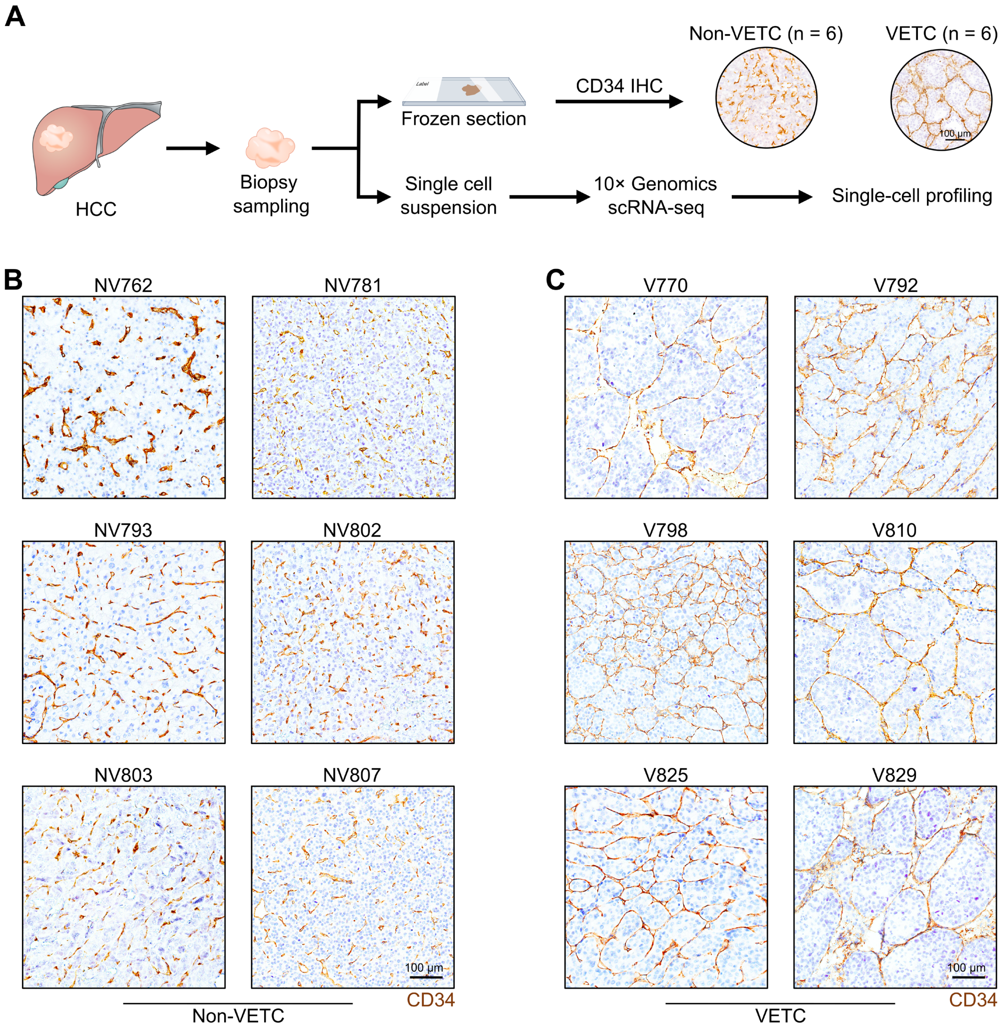

Supplemental Figure 1. Workflow of scRNA-seq and immunohistochemical staining for vessels in Non-VETC and VETC HCCs. (A) Schematic diagram of the experimental workflow. Twelve HCC samples (6 VETC and 6 Non-VETC HCCs) were subjected to scRNA-seq analysis. **(B-C)** Representative images of CD34 staining in Non-VETC and VETC HCC tissues used for scRNA-seq. Scale bar, 100 μm.

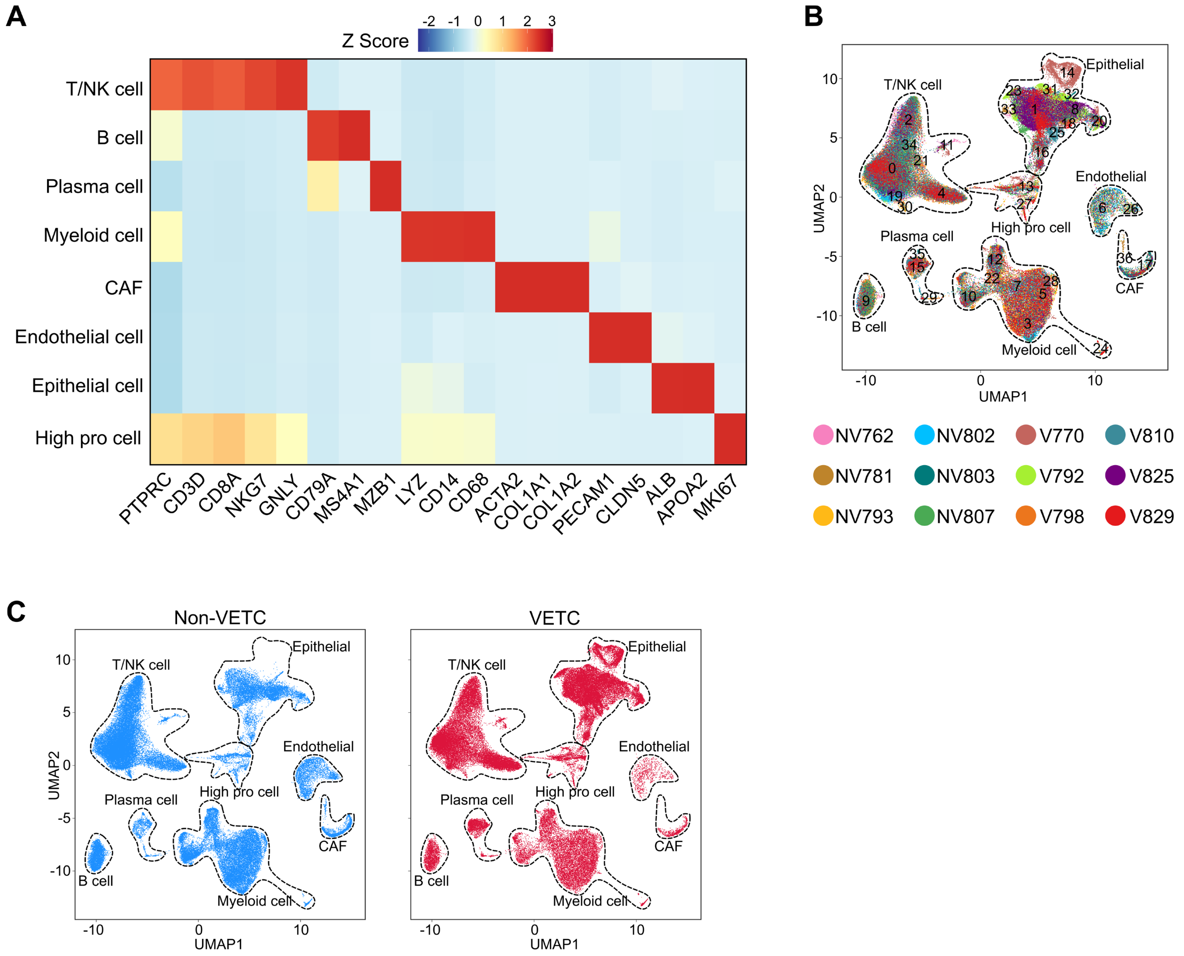

Supplemental Figure 2. scRNA-seq analysis revealed eight major cell clusters in HCC tissues. (A) Heatmap illustrated the RNA levels of marker genes for eight major cell clusters. Blue to red shading indicates low to high gene expression. **(B)** UMAP plot illustrated cell clusters in each tissue. **(C)** UMAP plot showed major cell clusters in VETC and Non-VETC HCCs. For (B and C), each dot represents a single cell.

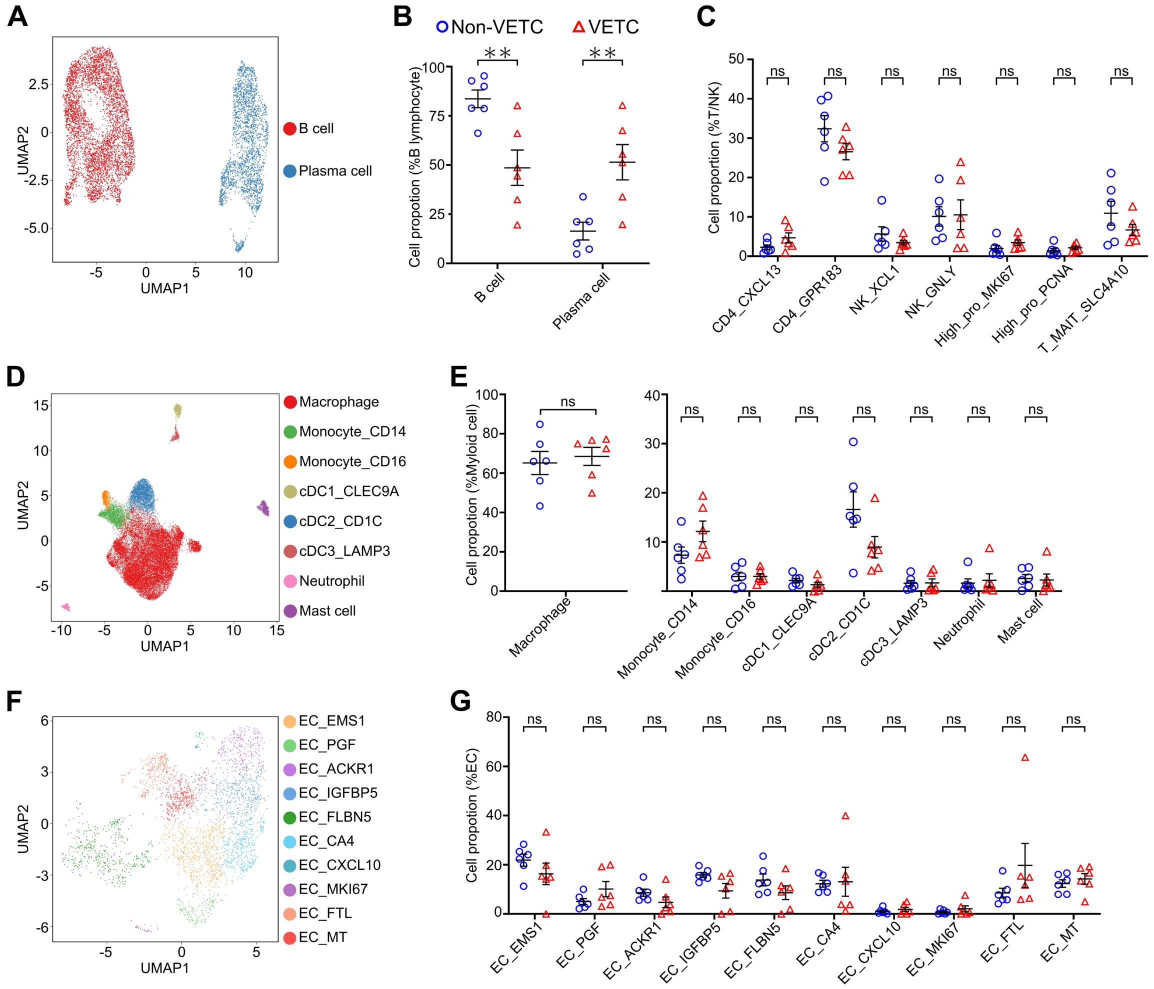

Supplemental Figure 3. Comparison of cell clusters in Non-VETC and VETC HCC tissues based on scRNA-seq data. (A-C) UMAP visualization and proportions of B lymphocyte (A and B), T and NK clusters (C). **(D and E)** UMAP visualization (D) and proportions (E) of myeloid cell clusters. **(F and G)** UMAP visualization (F) and proportions (G) of endothelial cell clusters. For (A, D, F), each dot represents a single cell. Non-VETC HCCs, n = 6 (B, C, E, G); VETC HCCs, n = 6 (B, C, E, G). Data are shown as mean ± SEM. ns, not significant; $^{**}P < 0.01$, by two-tailed Student's *t* test (B, C, E, G).

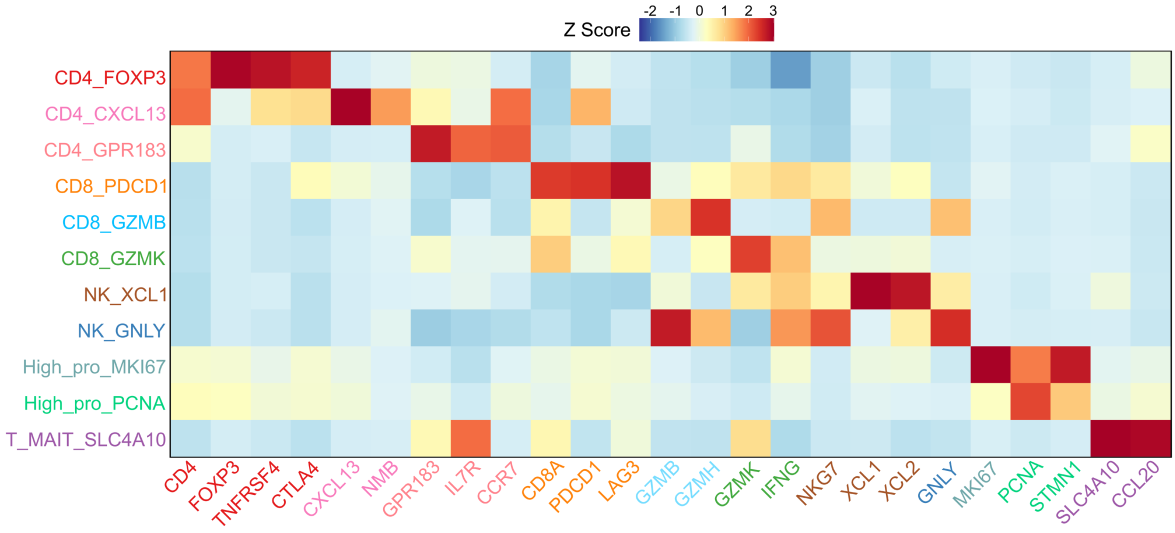

Supplemental Figure 4. scRNA-seq identifies T and NK cell clusters in HCC tissues. Heatmap showed the RNA levels of marker genes in T and NK cell clusters. Blue to red shading indicates low to high gene expression.

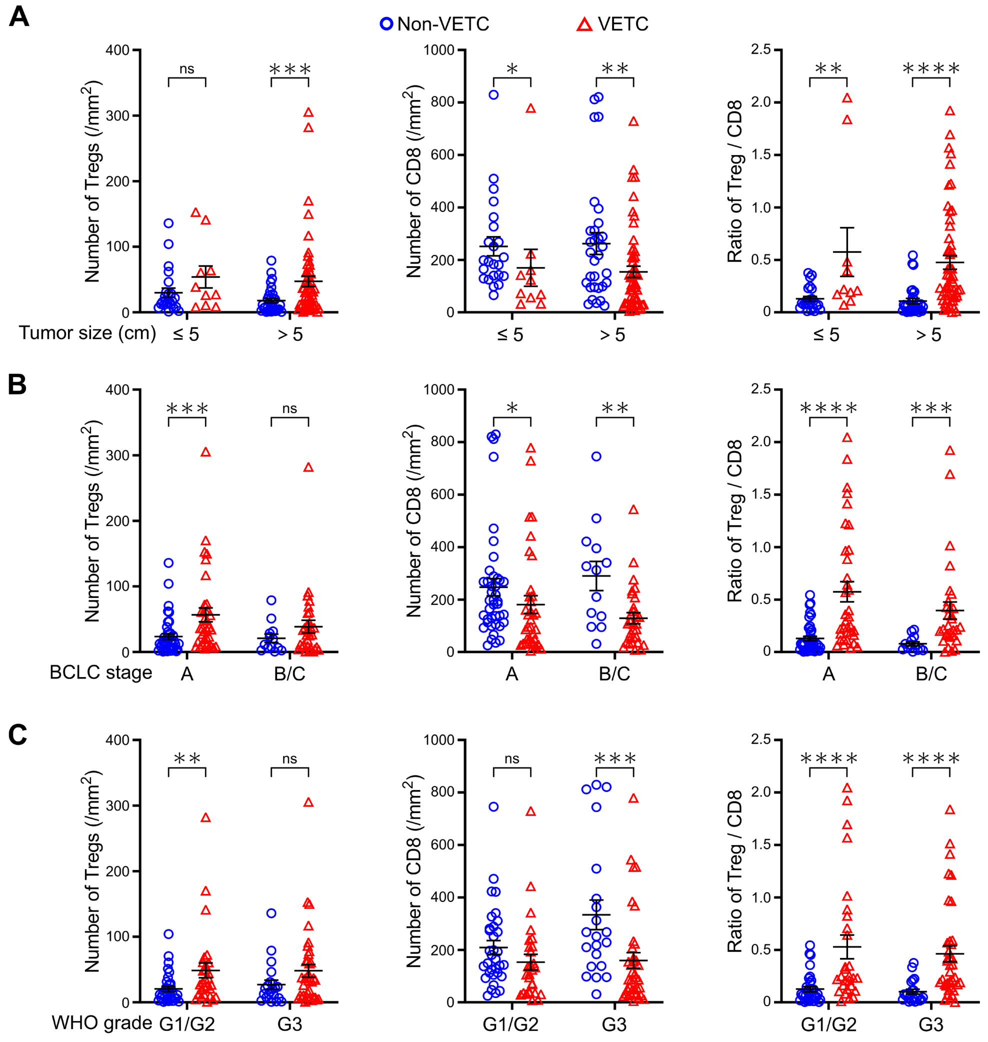

Supplemental Figure 5. Stratified analysis of Treg and CD8$^+$ T cell infiltration in the tumor tissues of HCC subgroups. (A) Stratified analysis by tumor size. (**B**) Stratified analysis by BCLC stage. (**C**) Stratified analysis by World Health Organization grading system (WHO grade). For (A-C), Non-VETC HCCs, n = 54; VETC HCCs, n = 65. In (A), stratified by tumor size: for tumor size ≤ 5 cm, Non-VETC HCCs, n = 23; VETC HCCs, n = 10; for tumor size > 5 cm, Non-VETC HCCs, n = 31; VETC HCCs, n = 55. In (B), stratified by BCLC stage: for BCLC A stage, Non-VETC HCCs, n = 41; VETC HCCs, n = 35; for BCLC B/C stage, Non-VETC HCCs, n = 13; VETC HCCs, n = 30. In (C), stratified by WHO grade: for WHO G1/G2 grade, Non-VETC HCCs, n = 33; VETC HCCs, n = 28; for WHO G3 grade, Non-VETC HCCs,

n = 21; VETC HCCs, n = 37.Data are shown as mean ± SEM. ns, not significant; *$P < 0.05$; **$P < 0.01$; ***$P < 0.001$; ****$P < 0.0001$, by Mann-Whitney *U* test (A-C).

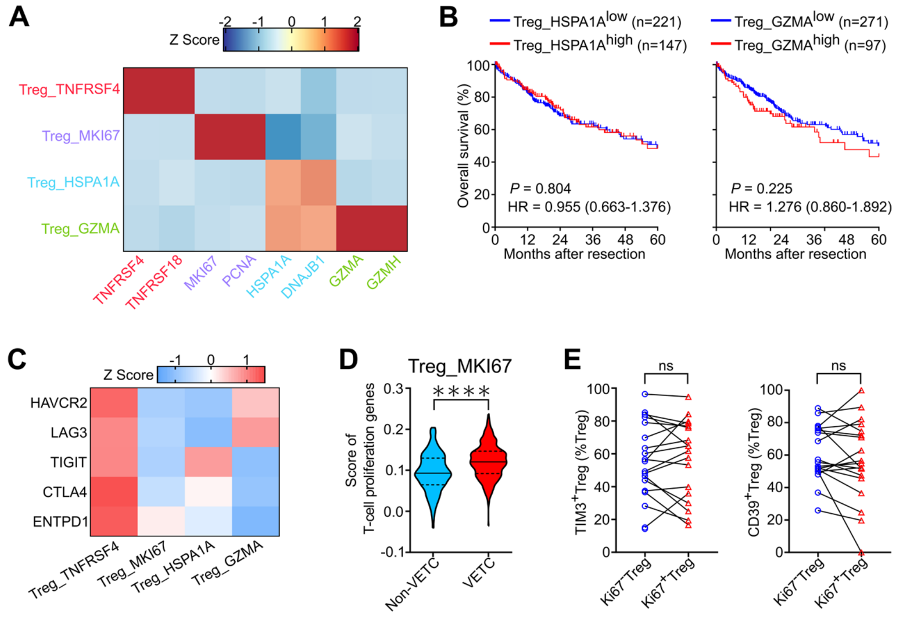

Supplemental Figure 6. Characterization of Treg clusters and its prognostic indications. **(A)** Heatmap showed RNA levels of marker genes for four Treg clusters. Blue to red shading indicates low to high gene expression. **(B)** Kaplan-Meier overall survival analysis. HCC patients (n = 368) from the TCGA cohort were stratified into high and low Treg_ HSPA1A (*left* panels) or Treg_GZMA (*right* panels) infiltration groups based on their signature genes in Supplemental Table 6. Infiltration levels were estimated by CIBERSORTx deconvolution on the RNA-seq data of HCC tissues from TCGA. **(C)** Heatmap illustrated the RNA levels of inhibitory checkpoint molecules across four Treg clusters. Blue to red shading indicates low to high gene expression. **(D)** The score of T cell proliferation genes in Treg_MIK67 cluster. Genes are listed in Supplementary Table 7. The central line of violin plot denotes median value (50th percentile), the upper and lower quartiles denote the 75th and 25th percentile, respectively. Non-VETC HCCs, n = 6; VETC HCCs, n = 6. **(E)** The ratio of $Ki67^+$Tregs and $Ki67^-$Tregs co-expressing TIM3 or CD39 (n = 19). Data are shown as mean ± SEM. ns, not significant; ****P < 0.0001, by log-rank tests (B), two-tailed Student’s *t* test (D and E).

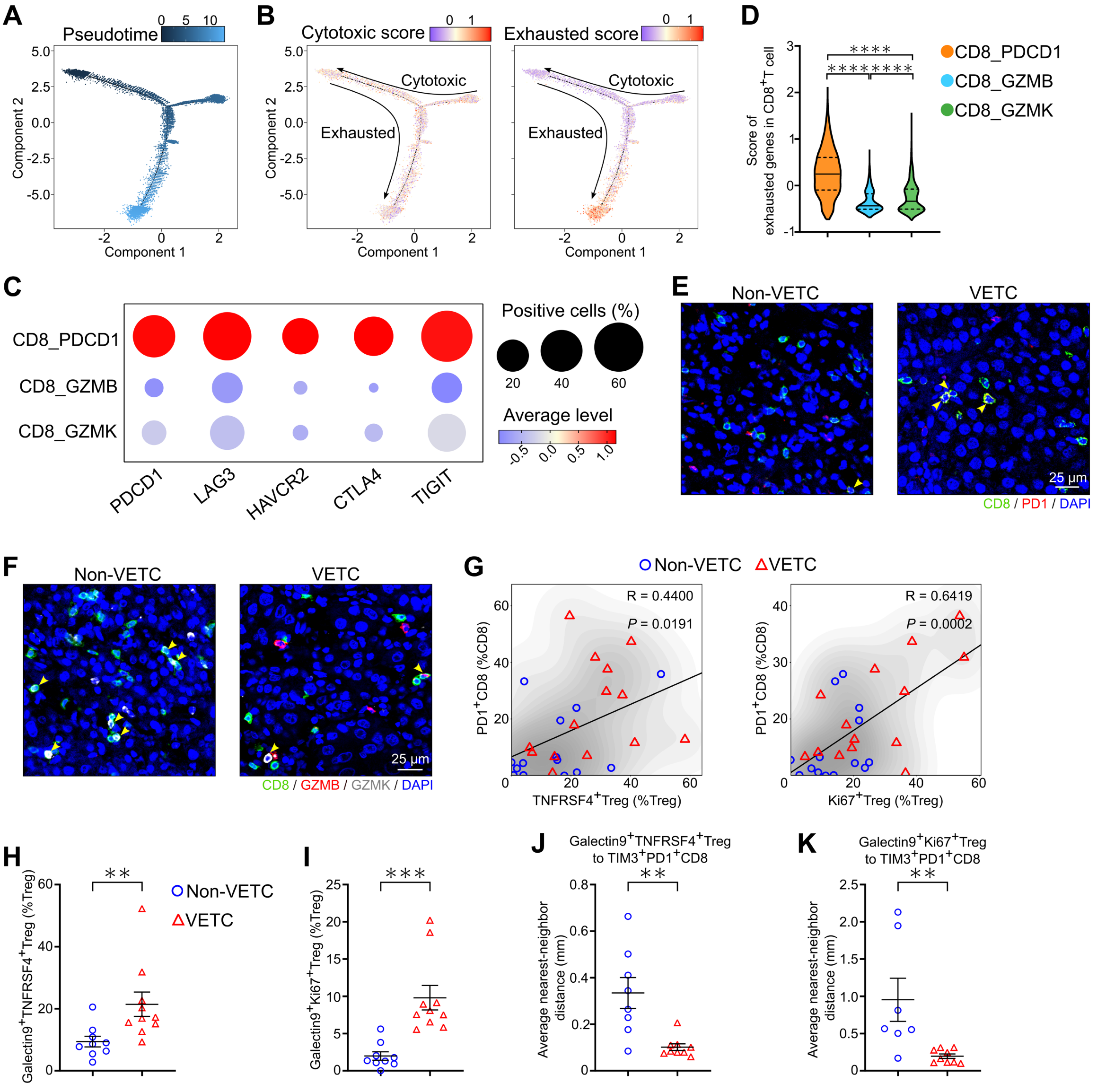

Supplemental Figure 7. Analysis of CD8^{+} T cell differentiation trajectory and the correlation between Treg and CD8^{+} T cell clusters. (A) Pseudotime trajectory analysis on CD8^{+} T cell differentiation based on gene expression and transition profiles. Cells are assigned pseudotime scores, dark to light blue shading indicates early to terminal states. **(B)** The cytotoxic score (*left* panel) and exhausted score (*right* panel) during CD8^{+} T cell differentiation trajectory. Purple to red shading indicates low to high score. **(C)** The levels of exhausted genes in CD8^{+} T cell clusters. Dot size indicates percent of positive cells, color intensity denotes average expression levels. **(D)** The score of exhausted genes in CD8^{+} T cell clusters. Genes are listed in Supplementary Table 9. The central line denotes median value (50th percentile), the

upper and lower quartiles denote the 75th and 25th percentile, respectively. **(E and F)** Representative images of multiplex immunofluorescent staining for PD1$^+$CD8$^+$ T cells (E) and GZMB$^+$ or GZMK$^+$CD8$^+$ T cells (F) in Non-VETC and VETC HCCs. Scale bar, 25 μm. **(G)** Positive correlation between the proportion of TNFRSF4$^+$Tregs (*left* panel) or Ki67$^+$Tregs (*right* panel) and the fraction of PD1$^+$CD8$^+$ T cells in HCC tissues (n = 28). R represents the Pearson correlation coefficient. **(H and I)** Higher proportions of Galectin9$^+$TNFRSF4$^+$Tregs (H) and Galectin9$^+$Ki67$^+$Tregs (I) in VETC HCCs compared with Non-VETC HCCs. **(J and K)** Closer spatial proximity between Galectin9$^+$ TNFRSF4$^+$Tregs (J) or Galectin9$^+$Ki67$^+$Tregs (K) and TIM3$^+$PD1$^+$CD8$^+$ T cells in VETC HCCs compared with Non-VETC HCCs. Non-VETC HCCs, n = 14 (G), 9 (H, I), 8 (J), 7 (K); VETC HCCs, n= 14 (G), 10 (H, I), 9 (J, K). Data are shown as mean ± SEM. $^{**}P < 0.01$; $^{***}P < 0.001$; $^{****}P < 0.0001$, by one-way ANOVA followed by Tukey's test (D), Pearson's correlation test (G), Mann-Whitney *U* test (H-K).

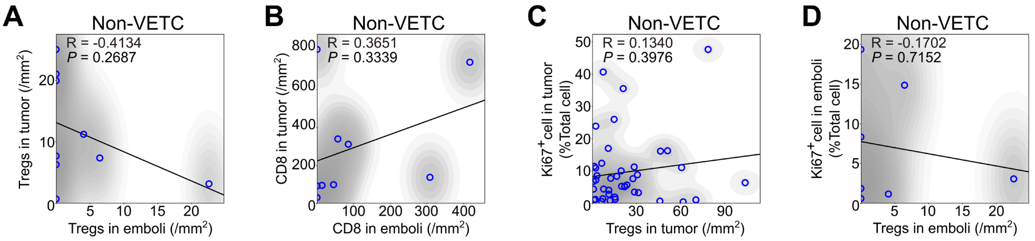

Supplemental Figure 8. The correlation analysis for cell clusters in primary tumors and tumor emboli of Non-VETC HCCs. (A-B) Correlation analysis of Treg (A) or $CD8^{+}$ T cell (B) number in primary tumors and tumor emboli (n = 9). **(C-D)** Association analysis between the number of Tregs and the fraction of $Ki67^{+}$ cells in primary tumors (C, n = 42) or tumor emboli (D, n = 7). For (A-D), R represents the Pearson correlation coefficient, Pearson's correlation test was applied.

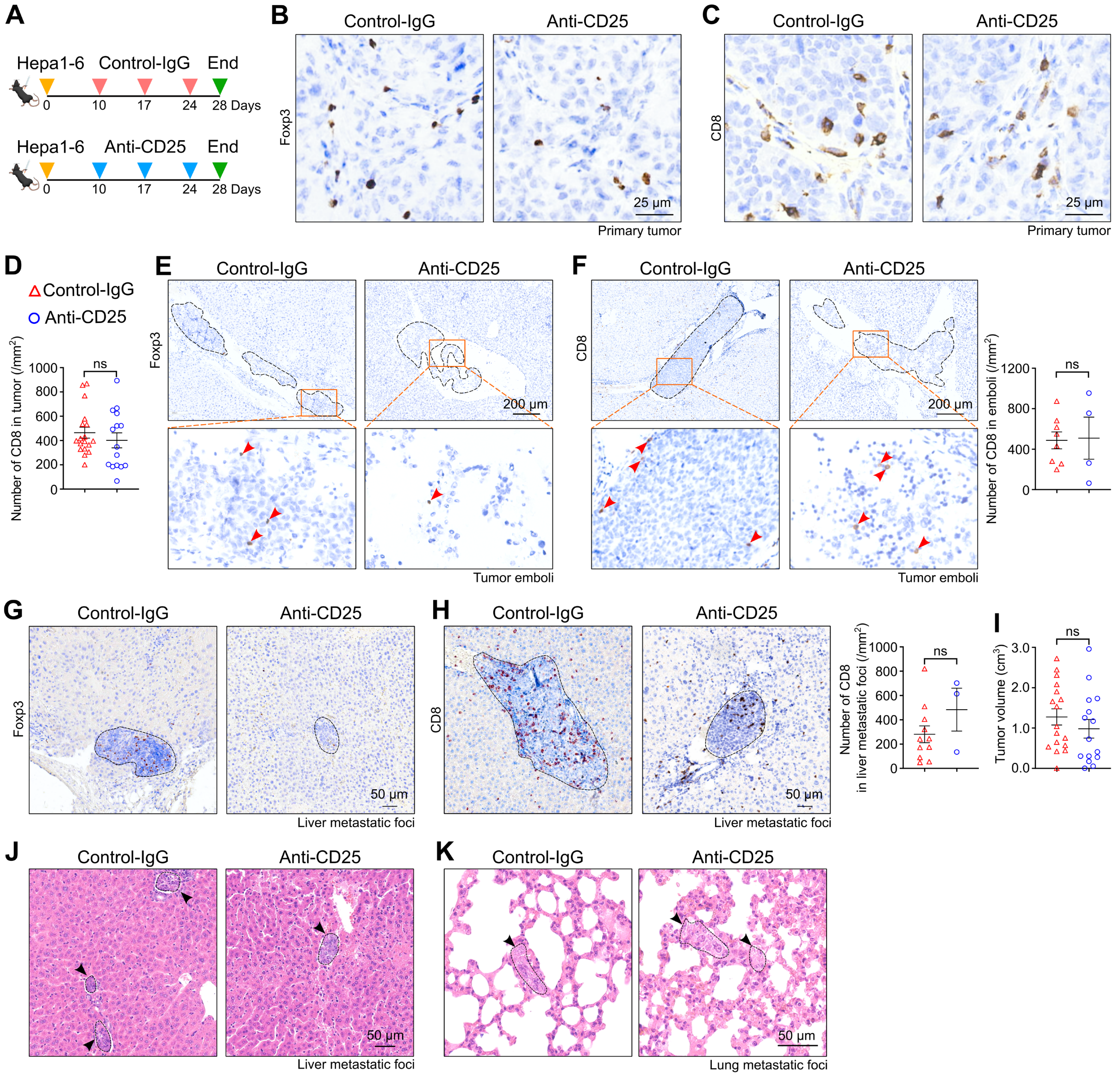

Supplemental Figure 9. Treatment with anti-CD25 antibody does not affect CD8$^+$ T cell number and tumor volume in Hepa1-6 allografts. (A) Schematic of Treg depletion by anti-CD25 in mouse liver orthotopic allograft model. **(B-D)** The effects of anti-CD25 antibody on the number of Tregs (B) and CD8$^+$T cells (C-D) in the primary site of Hepa1-6 allografts. Scale bar, 25 µm. **(E-F)** The roles of anti-CD25 antibody on the number of Tregs (E) and CD8$^+$ T cells (F) in the tumor emboli of Hepa1-6 allografts. Black dashed line outlines tumor embolus. Scale bar, 200 µm. **(G-H)** The effects of anti-CD25 antibody on the number of Tregs (G) and CD8$^+$ T cells (H) in the liver metastatic foci of Hepa1-6 allografts. Scale bar, 50 µm. **(I-K)** The influence of anti-CD25 antibody on tumor volume (I), liver (J) and lung (K) metastasis of

Hepa1-6 allografts. Scale bar, 50 μm. Control-IgG, n = 17 (D, I) or 8 (F) or 11 (H); Anti-CD25, n = 15 (D, I) or 4 (F) or 3 (H). Data are shown as mean ± SEM. ns, not significant, by Mann-Whitney *U* test (D), two-tailed Student's *t* test (F, H, I).

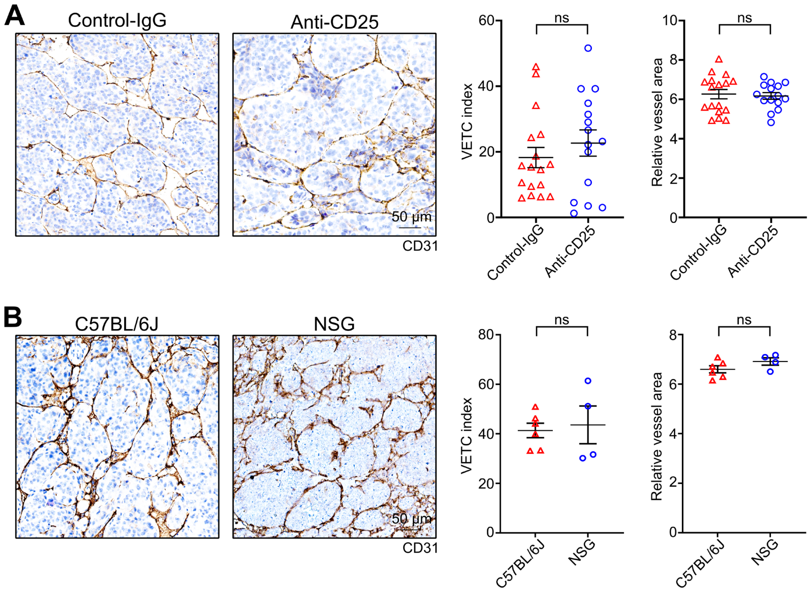

Supplemental Figure 10. Depletion of lymphocytes does not affect VETC formation. (A) Effects of anti-CD25 antibody on VETC pattern and vessel area in Hepa1-6 allografts. Control-IgG, n = 17; Anti-CD25, n = 15. **(B)** VETC pattern and vessel area in Hepa1-6 allografts from C57BL/6J and NSG mice. Scale bar, 50 µm. C57BL/6J, n = 6; NSG, n = 4. Data are shown as mean ± SEM. ns, not significant, by Mann-Whitney *U* test (A, *left* panel), two-tailed Student's *t* test (A, *right* panel; B).

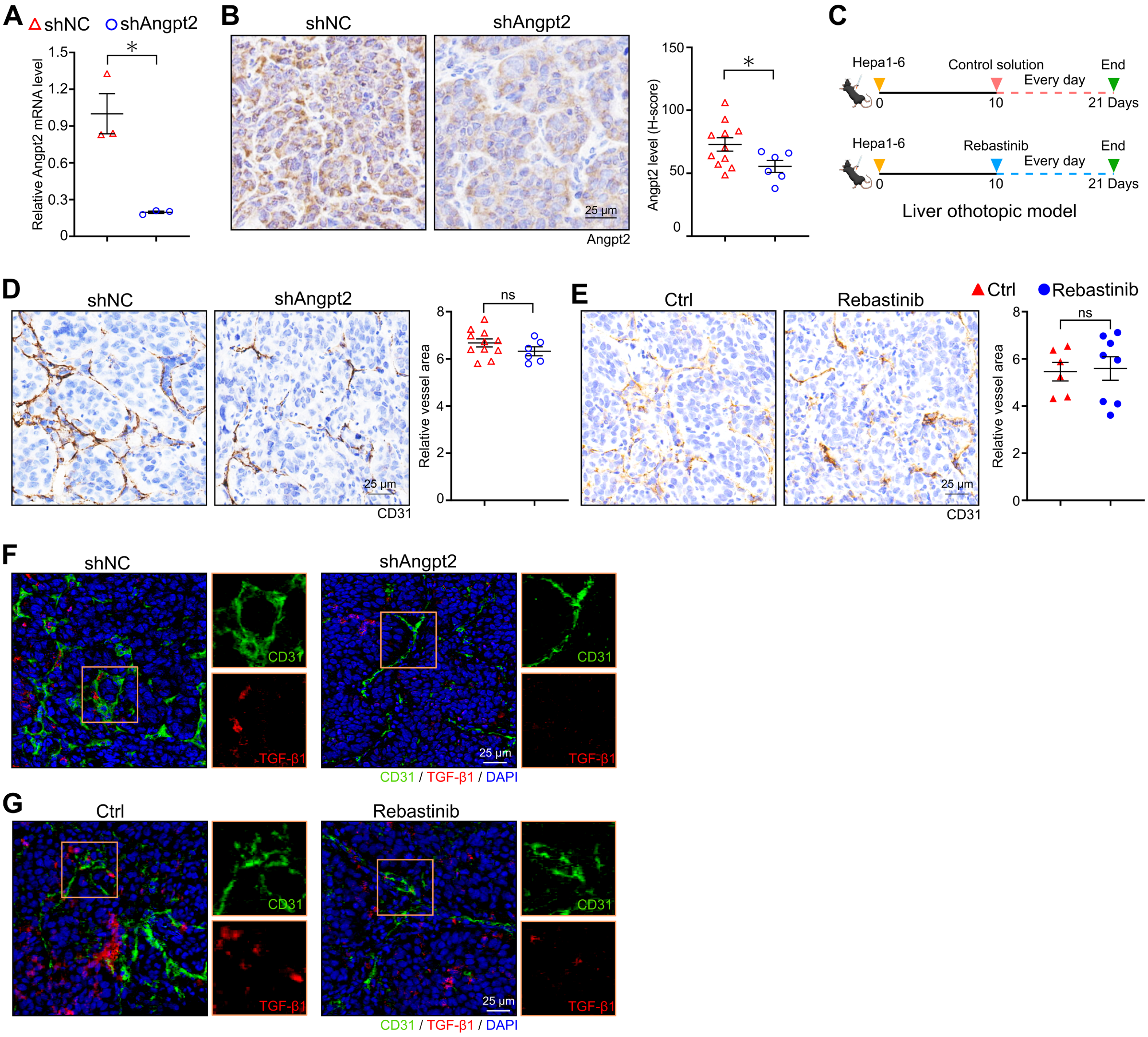

Supplemental Figure 11. Blocking the Angpt2/Tie2 signaling disrupts VETC formation and reduces endothelial TGF-β1 expression in Hepa1-6 allografts. (A) Validation of Angpt2 knockdown in Hepa1-6 cells. Data from three independent experiments (n = 3). **(B)** The Angpt2 levels in Hepa-shNC and Hepa-shAngpt2 allografts. **(C)** Schematic of Rebastinib treatment in mouse liver orthotopic allograft model. **(D and E)** VETC pattern and vessel area in Hepa-shAngpt2 allografts (D) and Rebastinib-treated Hepa1-6 allografts (E). **(F-G)** The TGF-β1 levels in the TECs of Hepa-shAngpt2 allografts (F) and Rebastinib-treated Hepa1-6 allografts (G). Scale bar, 25 μm. For (B-G), shNC, Hepa-shNC allografts (negative control, n = 11); shAngpt2, Hepa-shAngpt2 allografts n = 6; Ctrl, Hepa1-6 allografts treated with control solution (n = 6); Rebastinib, Hepa1-6 allografts treated with Rebastinib (Tie2 inhibitor, n = 8).

Data are shown as mean ± SEM. ns, not significant; *P < 0.05, by two-tailed Student's t test (A, B, D, E).

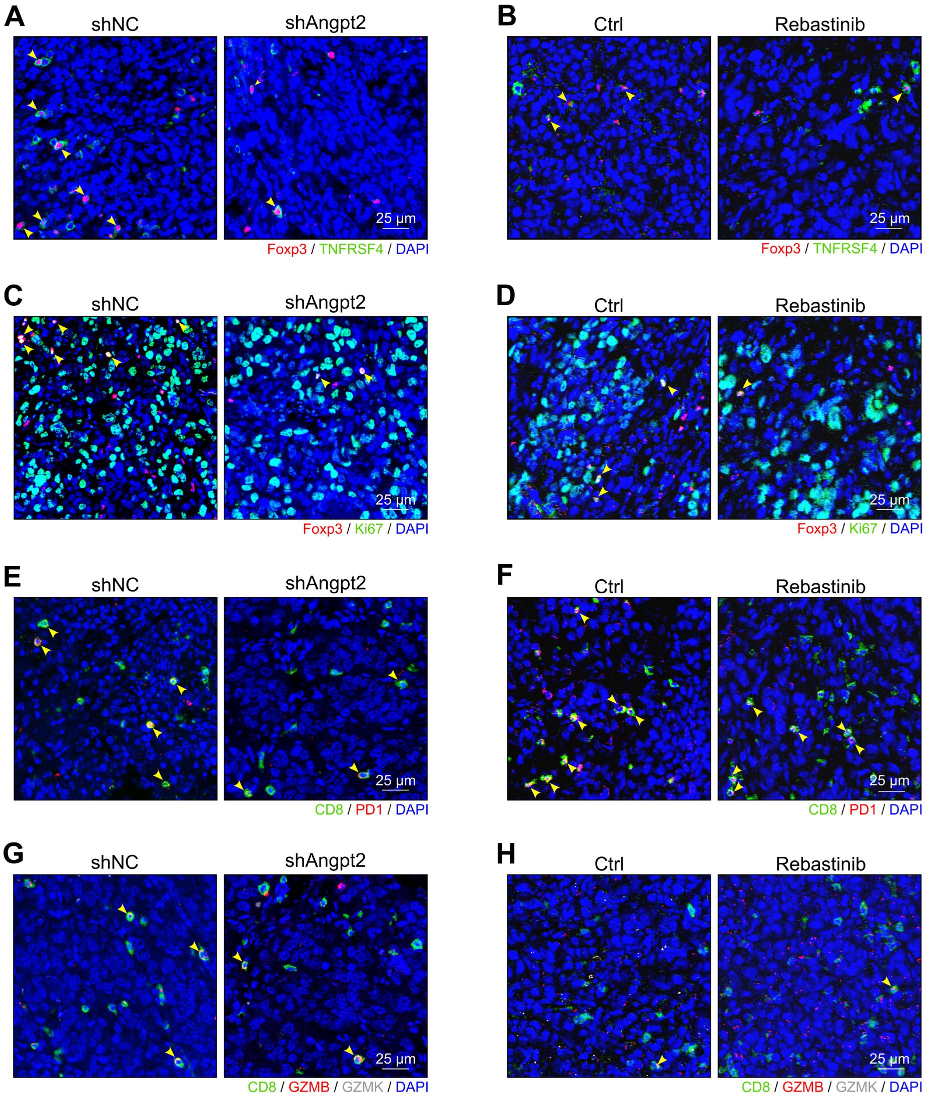

Supplemental Figure 12. Disruption of VETC formation by inhibiting the Angpt2/Tie2 signaling attenuates the immunosuppression in VETC tumor microenvironment. (A-D) Representative images of TNFRSF4$^+$ Tregs (A and B) and Ki67$^+$ Tregs (C and D) in Hepa-shAngpt2 (A and C) and Rebastinib-treated Hepa1-6 (B and D) allografts and their control allografts. **(E-H)** Representative images of PD1$^+$CD8$^+$ (E and F) and GZMB$^+$/GZMK$^+$CD8$^+$ (G and H) T cells in Hepa1-shAngpt2 (E and G) and Rebastinib-treated Hepa1-6 (F and H) allografts and their control allografts. Scale bar, 25 μm.

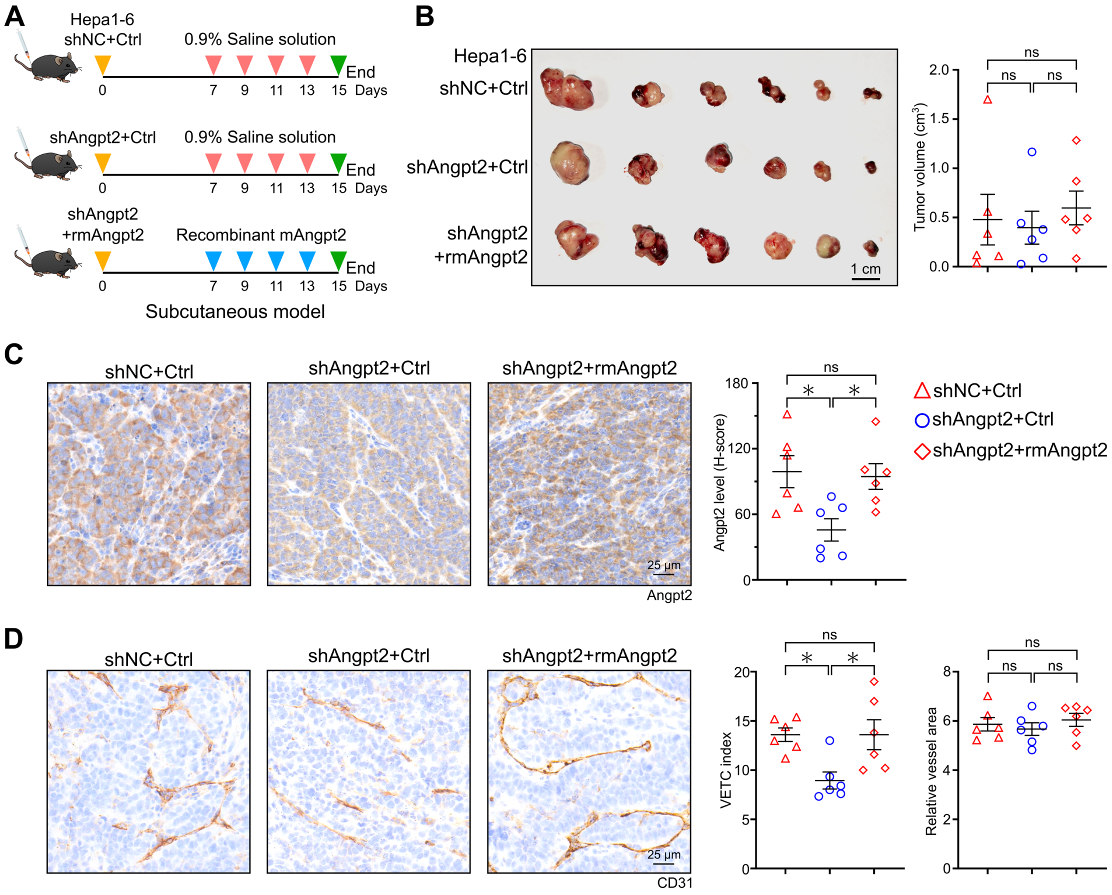

Supplemental Figure 13. Supplementation with recombinant Angpt2 rescues the VETC formation in Hepa-shAngpt2 allografts. (A) Schematic of Angpt2 rescue experiment in mouse subcutaneous allograft model. **(B)** Tumor growth following Angpt2 silencing and supplementation. Scale bar, 1 cm. **(C and D)** The effects of Angpt2 supplementation on Angpt2 level (C), VETC pattern and vessel area (D) in Hepa-shAngpt2 allografts. For (B-D), shNC+Ctrl, Hepa-shNC allografts treated with 0.9% saline (negative control, n = 6); shAngpt2+Ctrl, Hepa-shAngpt2 allografts treated with 0.9% saline (n = 6); shAngpt2+rmAngpt2, Hepa-shAngpt2 allografts injected with recombinant mouse Angpt2 (n = 6). Scale bar, 25 μm. Data are shown as mean ± SEM. ns, not significant; *$P < 0.05$, by one-way ANOVA followed by Tukey's test (B-D).

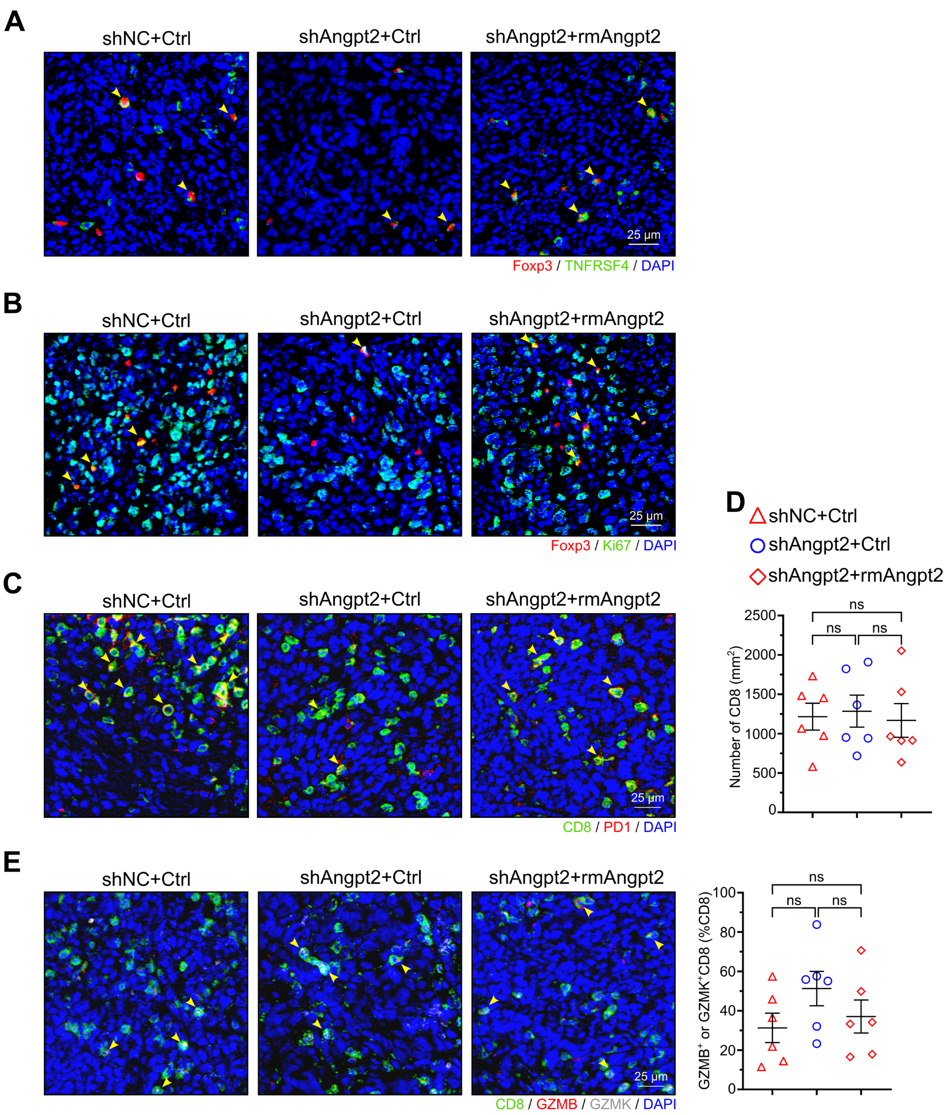

Supplemental Figure 14. Effects of Angpt2 supplementation on the characteristics of Tregs and CD8$^+$ T cells in subcutaneous Hepa1-6 allografts. (A-C) Representative images for TNFRSF4$^+$ Tregs (A), Ki67$^+$ Tregs (B), and PD1$^+$CD8$^+$ T cells (C). **(D and E)** The number of CD8$^+$T cells (D) and the proportion of GZMB$^+$ or GZMK$^+$CD8$^+$ T cells (E) in Hepa-shAngpt2 allografts without or with Angpt2 supplementation. For (D and E), shNC+Ctrl, Hepa-shNC allografts treated with 0.9% saline (negative control, n = 6); shAngpt2+Ctrl, Hepa-shAngpt2 allografts treated with 0.9% saline (n = 6); shAngpt2+rmAngpt2, Hepa-shAngpt2 allografts treated with recombinant Angpt2 (n = 6). Scale bar, 25 μm. Data are shown as mean ± SEM. ns, not significant, one-way ANOVA followed by Tukey's test (D and E).

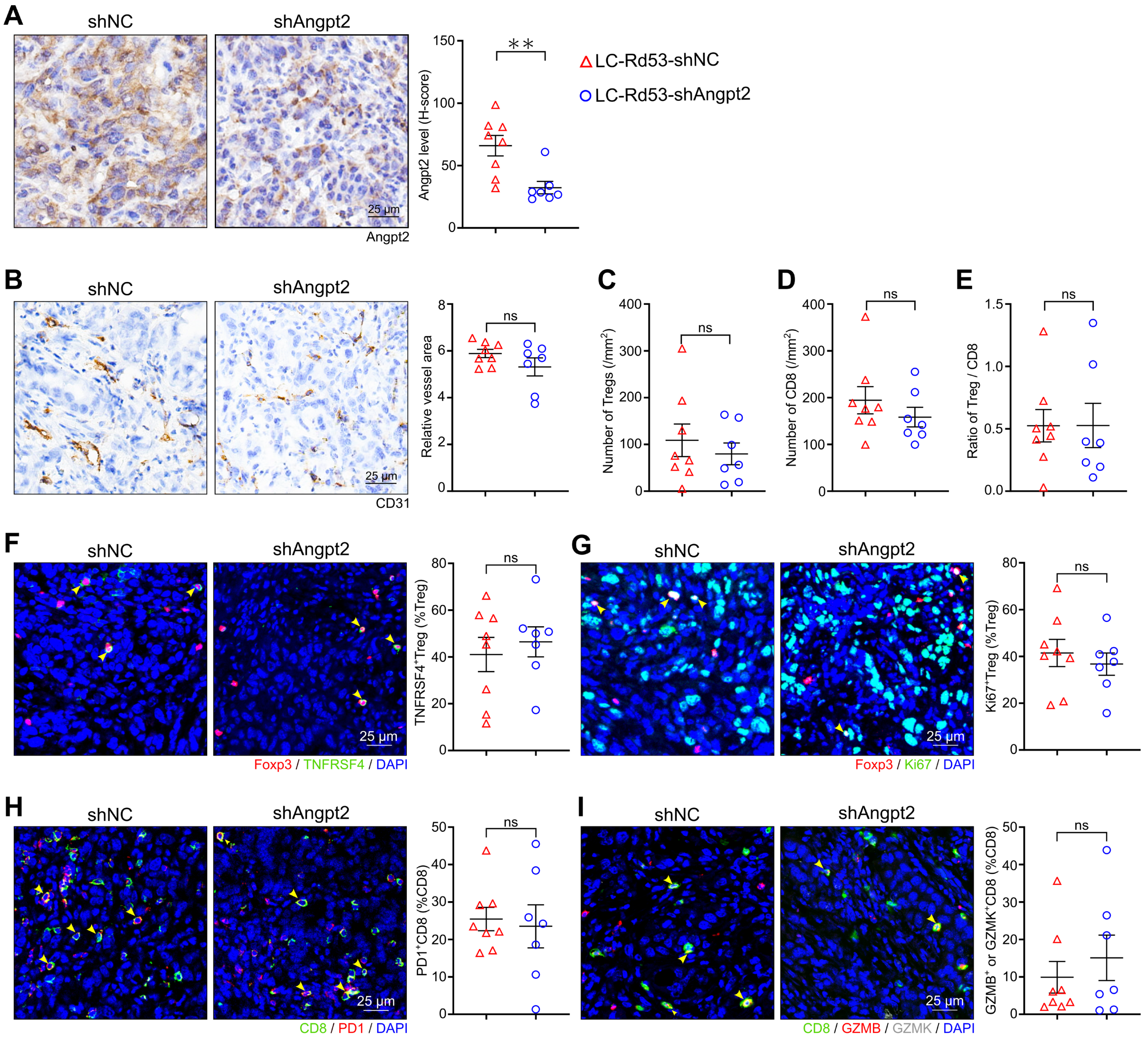

Supplemental Figure 15. Silencing Angpt2 does not affect the vascular pattern and the characteristics of Tregs and CD8⁺ T cells in the liver orthotopic allografts of Non-VETC hepatoma cells. (A) Validation of Angpt2 knockdown in LC-Rd53 allografts. **(B)** Relative vessel area in LC-Rd53 allografts. **(C–E)** The number of Tregs (C), CD8$^+$ T cells (D) and the ratio of Tregs/CD8$^+$ T cells (E) in LC-Rd53-shAngpt2 allografts. **(F–I)** The proportions of TNFRSF4$^+$Tregs (F), Ki67$^+$Tregs (G), PD1$^+$CD8$^+$ T cells (H) and GZMB$^+$ or GZMK$^+$CD8$^+$ T cells (I). For (A-I), LC-Rd53-shNC, LC-Rd53-shNC allografts (n = 8); LC-Rd53-shAngpt2, LC-Rd53-shAngpt2 allografts (n = 7). Scale bar, 25 μm. Data are shown as mean ± SEM. ns, not significant; ***P* < 0.01, by Mann-Whitney *U* test (A, I), two-tailed Student's *t* test (B-H).
